# An *in vitro* model of the epithelial airway reveals a key function for EHF in lung homeostasis and disease

**DOI:** 10.1242/dmm.052106

**Published:** 2025-07-01

**Authors:** Laetitia Pinte, Marta Vila-Gonzalez, Eleanor C. Williams, Erika Causa, Ricardo Fradique, Tekle Pauzaite, Charlotte Passemar, Silvia Becca, Christopher Gribben, Shiqi Ye, Maha Al-Thani, Fabian Bachinger, Floris J. M. Roos, James A. Nathan, Irina Mohorianu, Andres Floto, Pietro Cicuta, Ludovic Vallier

**Affiliations:** ^1^ Wellcome - MRC Cambridge Stem Cell Institute, University of Cambridge, Cambridge CB2 0AW, UK; ^2^ Cavendish Laboratory, University of Cambridge, Cambridge CB3 0HE, UK; ^3^ Cambridge Institute of Therapeutic and Infectious Disease, University of Cambridge, Cambridge CB2 0AW, UK; ^4^ MRC Laboratory of Molecular Biology, University of Cambridge, Cambridge CB2 0AW, UK; ^5^ Department of Surgery, University of Cambridge and NIHR Cambridge Biomedical Research Centre, Cambridge CB2 0AW, UK; ^6^ Department of Surgery, Cambridge University Hospitals NHS Foundation Trust, Cambridge CB2 0QQ, UK; ^7^ Stroke Research Group, Department of Clinical Neurosciences, University of Cambridge, Cambridge CB2 0QQ, UK; ^8^ Berlin Institute of Health, Center for Regenerative Therapies, Augustenburger Platz 1, 13353 Berlin, Germany

**Keywords:** Stem cell biology, Induced pluripotent stem cells, Lung airway pathophysiology, Disease modelling

## Abstract

In the lung airways, multiple cell types facilitate airflow to alveoli, clearing out debris, particles and pathogens. These vital processes are impeded in chronic inflammatory respiratory diseases, in which the epithelium typically suffers from inflammation, infections and hypoxia. An increasing body of evidence highlights the critical role of modifier genes in responses and resistance against these pathogenic processes. Here, we sought to study the transcription factor *EHF*, suggested by previous studies as a putative modifier gene, yet its functional role remains ambiguous. To explore this question, we knocked out EHF in human induced pluripotent stem cell-derived lung cells and examined the subsequent phenotypic and functional impacts. Loss of EHF enhanced cystic fibrosis transmembrane conductance regulator activity, led to transcriptomic changes in basal cells, increased transepithelial electrical resistance and reduced HIF-1α-mediated response to hypoxia. Here, we show that variation in *EHF* expression can impact lung diseases through several mechanisms, thereby highlighting prospects for novel therapies.

## Teaser

**Editor's choice:** In human airway epithelial cells, the loss of EHF protein enhances CFTR activity, leads to transcriptomic changes in basal cells, increases transepithelial electrical resistance and reduces HIF-1α-mediated response to hypoxia.

## INTRODUCTION

The airways are constantly exposed to exogenous particles and microorganisms found in the air we breathe, and airway epithelial cells act as a defence barrier against these agents through multiple mechanisms, including mucociliary clearance. Mucus traps inhaled debris, particles and pathogens, and is then mechanically expelled from the airways thanks to the orchestrated movement of cilia on multiciliated cells (MCCs). This vital process is hindered in chronic inflammatory respiratory diseases, such as chronic obstructive pulmonary disease (COPD), asthma, cystic fibrosis (CF) [in which CF transmembrane conductance regulator (CFTR) is impaired], or respiratory infections. In these conditions, pathological processes encompass chronic inflammation, infections and fibrosis. In addition, recent studies have shown that airway hypoxia is a prevalent consequence of chronic respiratory diseases, notably in muco-obstructive diseases such as coronavirus disease 2019 (COVID-19) or CF (Zhang et al., 2022 preprint; Liu et al., 2020; Montgomery et al., 2017).

Of particular interest, CF is an autosomal recessive condition caused by mutations in the *CFTR* gene, which encodes the CFTR protein. This protein acts as a chloride ion channel, regulating salt and water transport across the membrane of various epithelial cells. Mutations that lead to an absent or dysfunctional CFTR protein disrupt ion transport, resulting in the accumulation of dehydrated, viscous mucus on epithelial surfaces. Although CF affects multiple organs, most morbidity and mortality are attributed to cystic fibrosis lung disease (CFLD). In CFLD, mucociliary clearance is impaired, leading to chronic inflammation, persistent bacterial infections, airway remodelling and, eventually, bronchiectasis. Interestingly, *CFTR* mutations explain only about half of the severity of CFLD, suggesting the involvement of additional factors, including other genes and potential genetic modifiers.

Genome-wide association studies (GWAS) have uncovered loci of potential gene modifiers (Cutting, 2015). Notably, several GWAS have identified the epithelia-specific transcription factor E26 transformation-specific homologous factor (*EHF*) as a potential modifier gene associated with CF (Corvol et al., 2015; Polineni et al., 2018). Initially, EHF was linked to oncological processes in human (Zhou et al., 2021; Liu et al., 2019; Oyelakin et al., 2022; Albino et al., 2012). However, recent studies indicate that the knockdown of *EHF* (by siRNA) in Calu-3 lung adenocarcinoma cells and human bronchial epithelial cells (HBECs) affected the expression of genes and proteins involved in wound repair, cell differentiation, ion channel expression and immune response (Fossum et al., 2014, 2017; Mutolo et al., 2018). A first study showed that the knockdown of EHF in Calu-3 cells disrupted epithelial barrier maintenance, delayed wound healing and altered inflammatory responses (Fossum et al., 2014). In a second study using HBECs, reduced EHF levels affected several key processes: it altered the secretion of neutrophil chemokines, impeded wound closure in HBECs and increased the expression of the SAM pointed domain-containing ETS transcription factor, a transcription factor known to contribute to goblet cell hyperplasia (Fossum et al., 2017). These studies provided significant insights into the potential involvement of EHF in the lung airways, yet the precise function of EHF in pathological processes, particularly those occurring in chronic inflammatory respiratory diseases, remains unclear. Here, we investigated the functional effects of EHF loss in our *in vitro* lung airway cell model, focusing on pathological processes such as infection and hypoxia.

In this work, we first explored the expression of *EHF* across human lung airway epithelial cells (AECs) by single-cell RNA sequencing (scRNAseq). *EHF* was expressed across all AECs, with the highest expression in basal cells and FOXN4-expressing cells. Second, we sought to explore the function of EHF in lung cells using an *in vitro* model for the airway epithelium based on human induced pluripotent stem cells (hiPSCs). CRISPR-based gene editing was used to introduce indels, which caused a frameshift leading to a premature termination codon and subsequent activation of nonsense-mediated decay (NMD). Two different hiPSC lines, each derived from a healthy subject, were used; one of these lines was a reporter line (NKX2.1^GFP^/TP63^mCherry^). To minimise off-target effects, different guide RNAs (gRNAs) were employed for editing, one for each cell line. Clones were selected based on their genotype: homozygous nonsense mutation (*EHF*^−/−^) and heterozygous nonsense mutation (*EHF*^+/−^) and non-edited (*EHF*^+/+^), serving as isogenic wild-type controls. These selected clones were then differentiated into AECs. Adapted from published protocols (Hawkins et al., 2021; Berical et al., 2022), our *in vitro* airway epithelial model included basal cells, deuterosomal cells, lung stem cells, SCGB3A2-expressing secretory cells and multi-ciliated cells (MCCs), which were cultured either as organoids or as air–liquid interface (ALI) cultures. In AECs, mRNA levels of *EHF* were found to be significantly decreased in *EHF*^−/−^ clones compared to the *EHF*^+/+^ clones by reverse transcription quantitative PCR (RTqPCR). Furthermore, EHF protein was found to be decreased in *EHF*^+/−^ clones and absent in *EHF*^−/−^ clones by western blotting, confirming the loss of EHF protein in *EHF*^−/−^ cells. AECs were then subjected to further assays: bacterial infection susceptibility and response to hypoxia. Loss of EHF impacted the expression of multiple genes involved in cell differentiation and response to stress, increased the transepithelial electrical resistance (TEER) in ALI cultures, increased CFTR activity in organoids and lowered HIF-1α response in hypoxia. Taken together, our results underline multifaceted functions of *EHF* in lung airway epithelial cells, both in homeostasis and under pathological or stress conditions, which highlights the potential importance of this gene in the context of respiratory disease.

## RESULTS

### In the lung airways, *EHF* is most highly expressed by basal and FOXN4-expressing cells

**Fig. 1.**
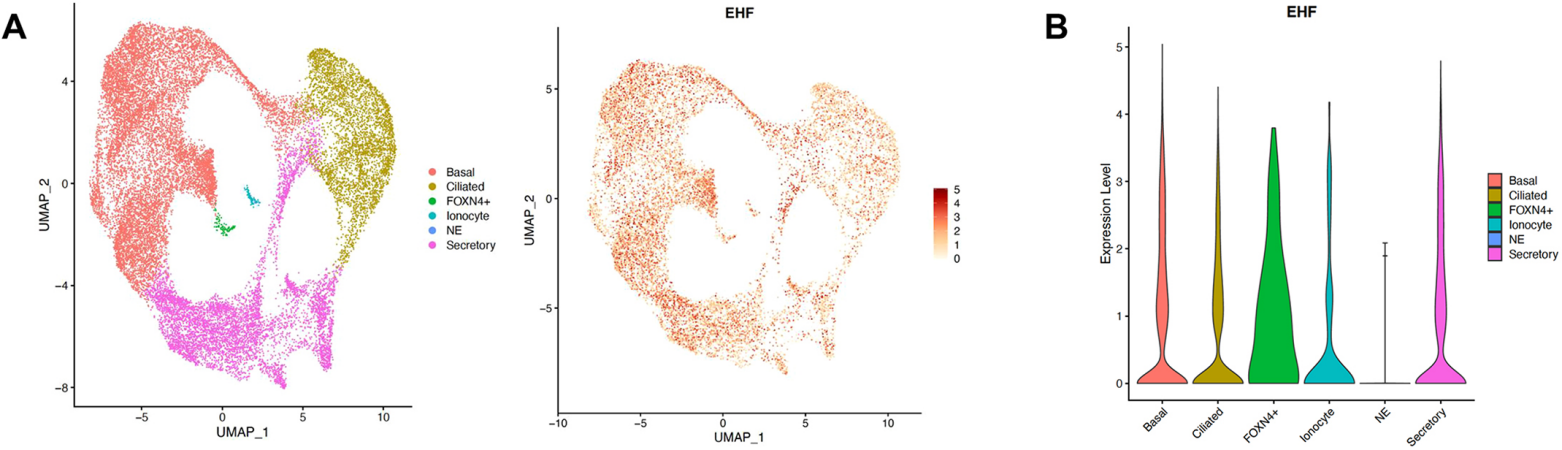
**EHF expression *in vivo*.** Uniform manifold approximation and projection (UMAP) visualisations of expression of EHF in primary bronchial epithelial cells. NE, pulmonary neuroendocrine cells.

To identify the array of specific cell types that express *EHF*, we assessed its expression in primary airway epithelial cells, using a publicly available scRNAseq dataset (Fig. 1A,B) (Carraro et al., 2021). *EHF* expression was detected across all cell types apart from pulmonary neuroendocrine cells (PNECs), with the highest levels observed in basal cells and FOXN4-expressing cells, called deuterosomal cells. We hypothesised that disruption of *EHF* expression would cause phenotypical and functional changes in airway epithelial cells, especially in basal and deuterosomal cells.

### hiPSC-based model generates AECs, including basal and deuterosomal cells

**Fig. 2.**
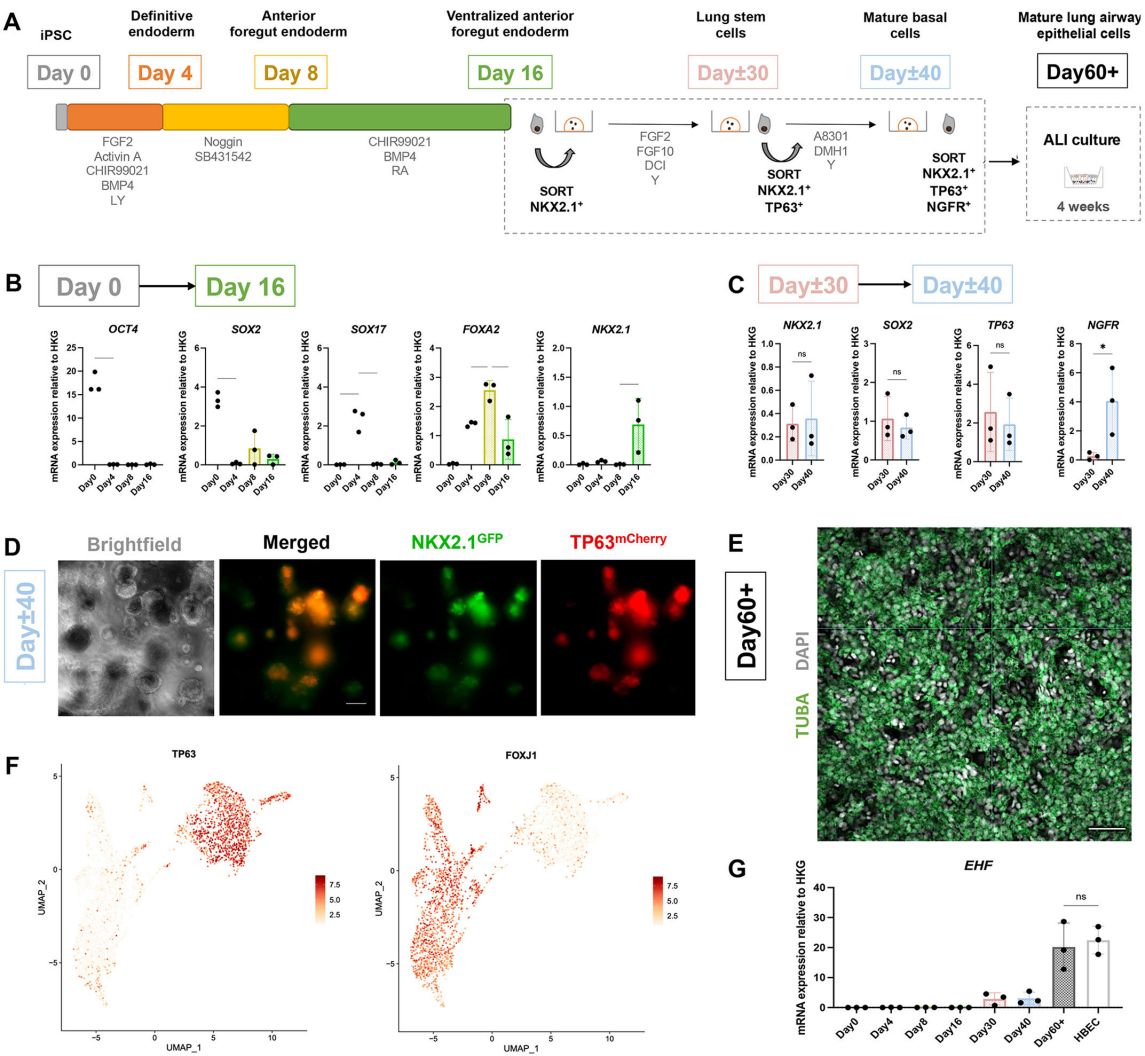
**Human induced pluripotent stem cell (hiPSC)-generated lung airway cells express EHF.** (A) Schematic of lung airway epithelial cell differentiation. ALI, air–liquid interface; iPSC, induced pluripotent stem cell. (B,C) Gene expression by reverse transcription quantitative PCR (RTqPCR), of cells at Day 0-16 (B) and Day 30-40 (C) relative to housekeeping genes. *n*=3 biological replicates of differentiations. Error bars represent s.d.; unpaired one-way ANOVA; ns, nonsignificant; *P<0.05. (D) Live imaging of Day 40 cells of reporters for NKX2.1^GFP^ and TP63^mCherry^ (scale bar: 100 μm). (E) Confocal microscopy of Day 60 ALI cultures immunolabeled with the antibodies indicated (scale bar: 50 μm). (F) UMAP visualisation of TP63 and FOXJ1 expression in Day 60 ALI culture cells. (G) Expression of *EHF* by RTqPCR, relative to housekeeping genes. *n*=3 biological replicates of differentiations and 2-week-old human bronchial epithelial cell (HBEC) cultures as ALI cultures (conditions outlined in Materials and Methods). Error bars represent s.d.; unpaired two-tailed Student's *t*-test of Day 60 versus HBECs; ns, nonsignificant.

To study EHF, we used a hiPSC-derived AEC model. We adapted three protocols to differentiate hiPSCs into AECs (Hawkins et al., 2021; Hannan et al., 2013; Konishi et al., 2016). First, hiPSCs were differentiated into definitive endoderm (Day 4) following a protocol published and validated by our group (Hannan et al., 2013), then induced into lung stem cells (Day 16) following Konishi et al. (2016) and finally to AECs (Day 40) following Hawkins et al. (2021) (Fig. 2A). Details can be found in the Materials and Methods section. This combination of protocols was chosen as it provided us with the most efficient and reproducible AECs phenotype (Vilà-González et al., 2024). The expression of key markers was observed through RTqPCR analysis: *SOX17*, *FOXA2* on Day 4 (definitive endoderm), *FOXA2* on Day 8 (anterior foregut endoderm) and *NKX2.1* (also known as *NKX2-1*) on Day 16 (lung stem cells) (Fig. 2B). Lung stem cells were then further matured into NKX2.1- and TP63-expressing basal cells (Fig. 2C), which, on Day 40, derived into NGFR-expressing basal cells (Fig. 2D). Day 40 basal cells were then sorted and seeded onto transwells to allow ALI cultures, in which differentiation into MCCs was shown by expression of α-tubulin (TUBA; Fig. 2E). To better characterise our ALI cultures, we performed scRNAseq analyses. Uniform manifold approximation and projection (UMAP) clustering outlined four populations: cycling basal cells (expressing *MKI67*, *TP63* and *KRT5*), basal cells (expressing *TP63* and *KRT5*), deuterosomal cells (expressing *FOXN4*, *CDC20B* and *SNTN*) and MCCs (expressing *FOXJ1* and *SNTN*) (Fig. 2F; Fig. S1). Moreover, we did not find mesenchymal cells, non-lung endodermal or distal lung populations, confirming that our protocol specifically produces proximal AECs, similar to previous publications (Hawkins et al., 2021). Importantly, to ensure the robustness of our findings, our study was performed using two different hiPSC lines with different genetic backgrounds (i.e. derived from different donors). These include a reporter NKX2.1^GFP^/TP63^mCherry^ hiPSC line that facilitates the isolation of populations of interest. The differentiation protocol for both lines remained unchanged, except for the cell sorting strategies. Briefly, lung cells generated from the standard hiPSC line were sorted twice [gating for cells with high CPM expression (CPM^HI^ cells) at Day 16 and NGFR^+^ cells at Day 40], while the reporter cells were sorted three times (NKX2.1^+^ at Day 16, TP63^+^/NKX2.1^+^ at Day 30 and TP63^+^/NKX2.1^+^/NGFR^+^ at Day 40) (see Materials and Methods section). Finally, we investigated the expression of *EHF* during *in vitro* differentiation. By RTqPCR, the expression of *EHF* was detectable after 30, 40 and 60 days of differentiation. Levels at Day 60 were comparable to levels in HBECs, suggesting that the final maturation is key to obtaining relevant *EHF* levels (Fig. 2G). Taken together, our results show that our culture system is suitable for studying the function of *EHF* in relevant EHF-expressing cell types such as basal and FOXN4-expressing cells.

### Editing of *EHF* in two hiPSC lines results in the absence of EHF protein

**Fig. 3.**
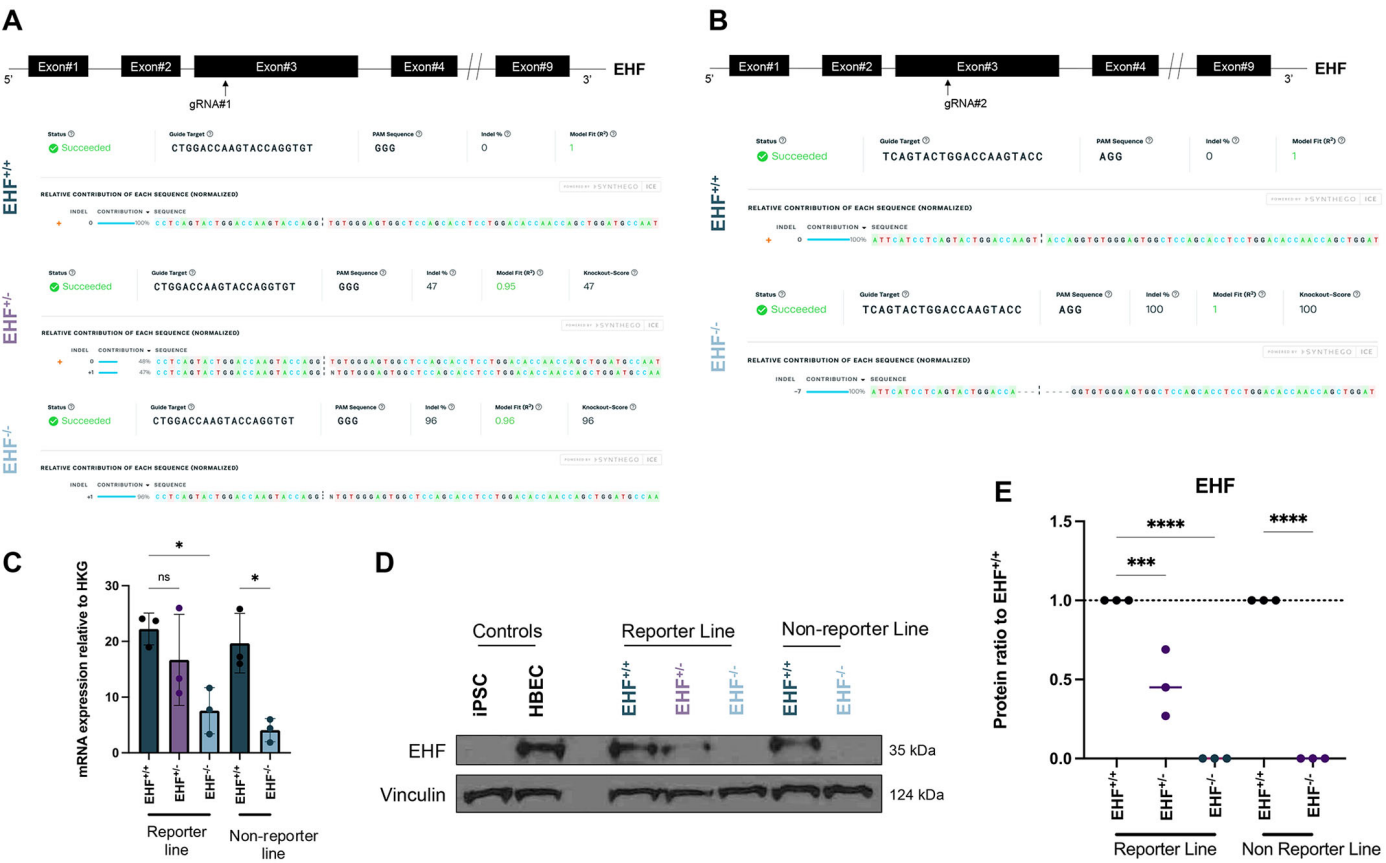
**Editing of EHF in hiPSC lines.** (A,B) InDel analysis of all edited clones used after Sanger sequencing, using ICE in a reporter iPSC line (A) and a non-reporter iPSC line (B). gRNA, guide RNA; PAM, protospacer adjacent motif. (C) Gene expression of *EHF* in Day 60 cells, assessed by RTqPCR and relative to housekeeping genes. *n*=3 biological replicates of differentiations for each hiPSC line. Error bars represent s.d.; paired one-way ANOVA; ns, nonsignificant; *P<0.05. (D) Protein expression of EHF in Day 60 cells, assessed by western blotting, in reporter and non-reporter lines. Controls used are an undifferentiated hiPSC line (iPSC) and HBECs derived from a primary cell sample. (E) Quantification of EHF per clones. *n*=3 experimental replicates. Error bars represent s.d.; paired, one-way ANOVA; ***P<0.0005, ****P<0.00005.

To eliminate EHF protein expression in our two hiPSC lines, we used CRISPR/Cas9-based genome editing to introduce indels in an early exon (exon 3), resulting in premature termination codon and inducing NMD. Each hiPSC line was targeted separately with different gRNAs (gRNA#1 and gRNA#2) to control for off-target effects and interline variability. hiPSC sublines were individually picked, expanded and genotyped. We selected edited clones in both iPSC lines [i.e. homozygous for nonsense mutation (*EHF*^−/−^) and heterozygous for nonsense mutation (*EHF*^+/−^) in the reporter line and homozygous for nonsense mutation (*EHF*^−/−^) in the non-reporter line] (Fig. 3A,B). Importantly, the non-edited clones (*EHF*^+/+^) underwent the same process and were used as isogenic controls (Fig. 3A,B). Based on their genotype, early stop codons are predicted to truncate the EHF protein, resulting in shorter proteins with molecular masses of 12.48 kDa and 8.99 kDa, instead of the full-length 35 kDa protein (Fig. S2A). Then, we differentiated each subline into airways cells to confirm the efficacy of our genome editing approach. Accordingly, the expression of *EHF* mRNA was significantly decreased in *EHF*^−/−^ clones after 60 days of differentiation compared to that in their wild-type counterparts (Fig. 3C), suggesting that our targeting strategy also decreases mRNA production, likely through NMD. Furthermore, we could not detect EHF protein by western blotting (Fig. 3D). Across experimental replicates, a significant decrease in EHF protein was observed in *EHF*^+/−^ cells compared to *EHF*^+/+^ cells (Fig. 3E). In addition, we did not find predicted truncated EHF proteins on the blots [both predicted truncated proteins should be detectable by western blotting using the anti-EHF polyclonal antibody we validated (Fig. S2A)], suggesting that the truncated EHF proteins had been degraded (Fig. S2B). These data confirmed that our gene editing was successful and resulted in the absence of EHF protein in hiPSC-derived AECs.

### Absence of EHF protein does not affect basal, SCGB3A2-expressing or ciliated cell production

**Fig. 4.**
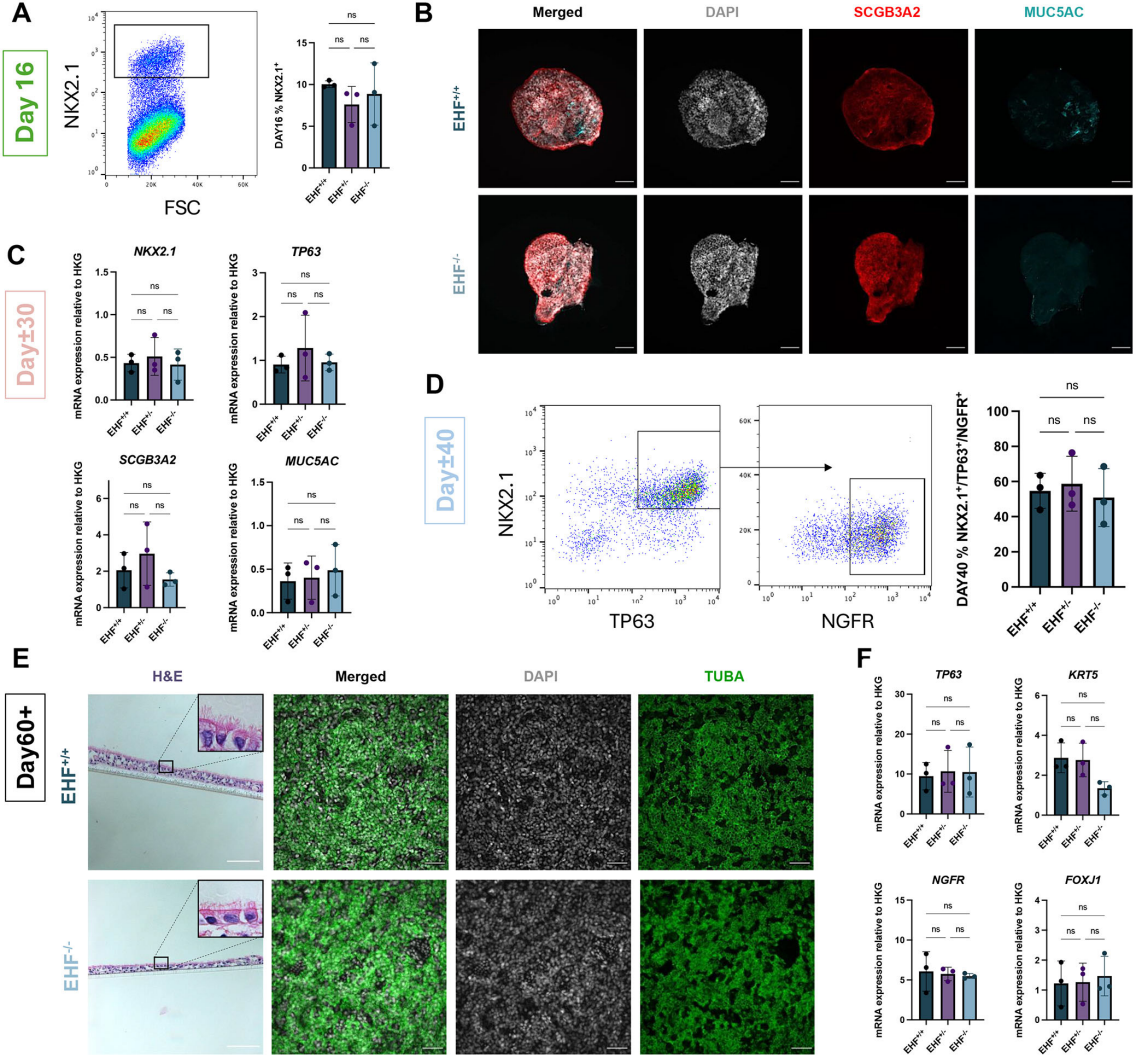
**Absence of EHF does not affect the generation of basal and multiciliated cells.** (A) Representative flow cytometry plot of NKX2.1^GFP^ reporter cells at Day 16. *n*=3 experimental replicates. Error bars represent s.d.; one-way ANOVA; ns, nonsignificant. (B) Images of Day 30 organoids from the reporter line by confocal microscopy immunolabeled with antibodies (scale bars: 100 μm). (C) Gene expression in Day 30 cells from the reporter line by RTqPCR and relative to housekeeping genes. *n*=3 experimental replicates. Error bars represent s.d.; one-way ANOVA; ns, nonsignificant. (D) Representative flow cytometry plot of NKX2.1^GFP^, TP63^mCherry^ and NGFR^BV421^ reporter cells at Day 40. *n*=3 experimental replicates. Error bars represent s.d.; one-way ANOVA; ns, nonsignificant. (E) Transverse section images of ALI cultures from the reporter line stained with Haematoxylin and Eosin (H&E) (scale bars: 100 μm) and immunolabeled with the indicated antibodies (scale bars: 50 μm). (F) Gene expression of Day 60 cells from the reporter line by RTqPCR and relative to housekeeping genes. *n*=3 experimental replicates. Error bars represent s.d.; one-way ANOVA; ns, nonsignificant.

Next, we explored the phenotype of our selected sublines after AECs differentiation. There were no significant differences in definitive endoderm, anterior foregut endoderm or lung stem cell markers in *EHF*^+/+^, *EHF*^+/−^ and *EHF*^−/−^ sublines (Vilà-González et al., 2024). Furthermore, the fraction of lung progenitors (NKX2.1^GFP^ or CPM^HI^) was similar by flow cytometry (Fig. 4A). On Day 30, there were no significant differences in *SCGB3A2*, *TP63* and *MUC5AC* expression by RTqPCR or immunocytochemistry between *EHF*^+/+^ and *EHF*^−/−^ AECs (Fig. 4B,C; Fig. S4A). Notably, we did not detect MUC5B-expressing goblet cells, MCCs or ionocytes in any of the subclones, consistent with our previous observations in non-targeted hiPSCs, as these cell types are not generated in our model (Fig. S4A). On Day 40, the proportions of mature basal cells (NKX2.1^+^/TP63^+^/NGFR^+^ in the reporter line or NGFR^+^ in the non-reporter line) were comparable, suggesting that the hiPSC lines display the same capacity of differentiation into basal cells as their wild-type counterpart (Fig. 4D). Finally, histological analyses revealed a polarised epithelium with apical cilia in all sublines, and immunofluorescence confirmed the presence of cilia (TUBA^+^) at the apical pole in similar proportions in *EHF*^+/+^ and *EHF*^−/−^ AECs after ALI differentiation (Fig. 4E). Moreover, *TP63*, *KRT5*, *NGFR* and *FOXJ1* levels were not significantly different when assessed by RTqPCR (Fig. 4F). Together, our results showed that absence of the full-length EHF protein does not impair hiPSC differentiation into lung stem cells, basal cells, SCGB3A2-expressing secretory cells and MCCs*.*

### Absence of EHF protein affects the transcriptome of hiPSC-derived AECs

**Fig. 5.**
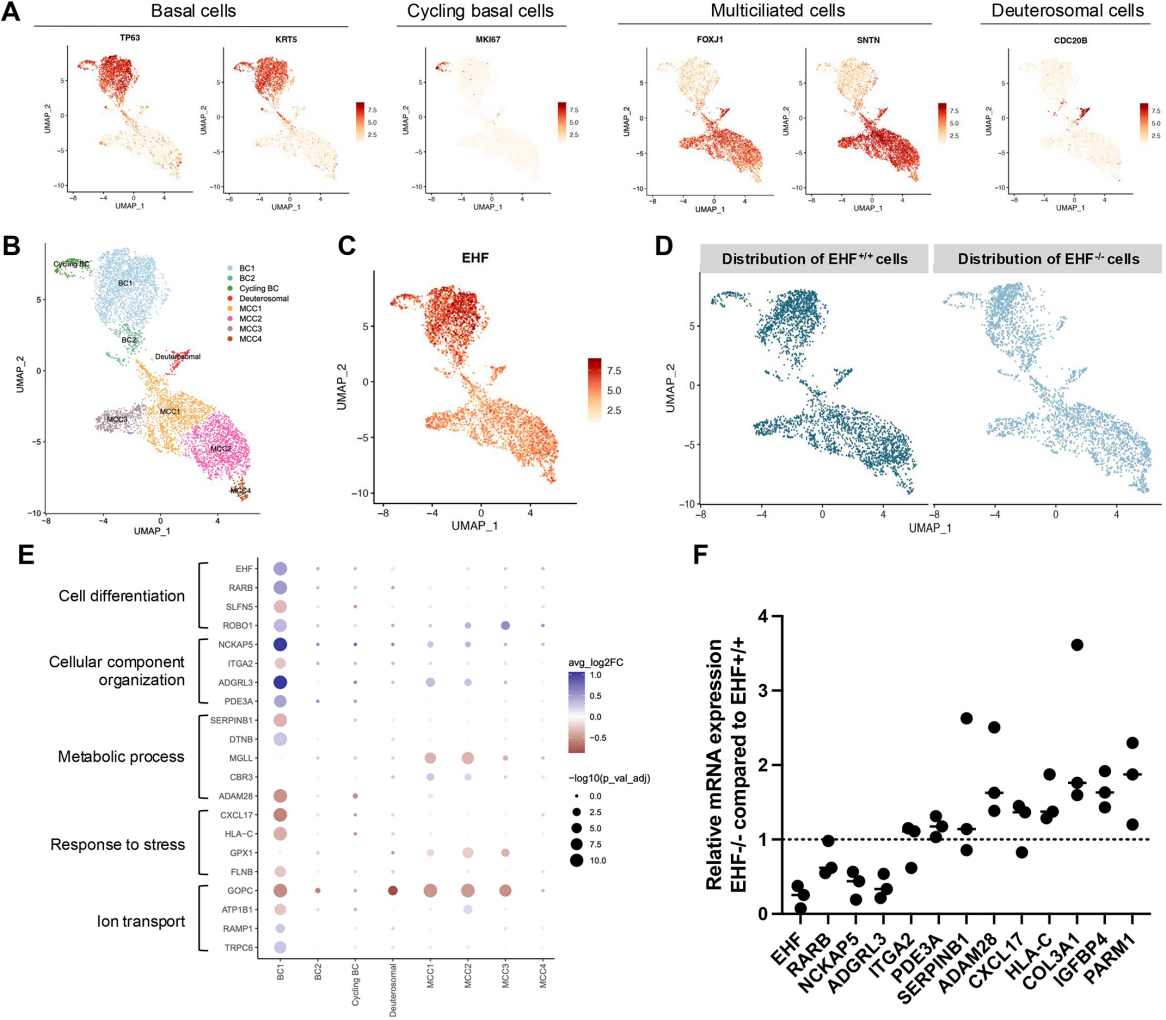
**Single-cell RNA sequencing (scRNAseq) reveals the impact of EHF knockout on the transcriptome in hiPSC-derived airway epithelial cells (AECs).** (A) UMAP visualisation of key genes in AEC differentiation. (B) UMAP visualisation of cells with EHF expression. (C) Repartition of *EHF*^+/+^ or *EHF*^−/−^ cells in UMAP visualisation. (D) UMAP visualisation of cells from additional lines coloured by cluster assignment. (E) Dot plots of differentially expressed genes in *EHF*^+/+^ compared to *EHF*^−/−^ samples and their associated terms. Browns represent genes expressed more in *EHF*^−/−^ cells than in *EHF*^+/+^ cells. (F) Relative expression of genes outlined by scRNAseq in *EHF*^−/−^ cells compared to *EHF*^+/+^ cells by RTqPCR.

To refine our phenotyping analyses, we performed scRNAseq on ALI-cultured AECs originating from *EHF*^+/+^ and *EHF*^−/−^ hiPSCs. Here, 5671 cells were explored, and UMAP analysis showed the presence of the four cell types (cycling basal cells, basal cells, deuterosomal cells and MCCs) in both *EHF*^+/+^ and *EHF*^−/−^ cells (Fig. 5A). Moreover, subcluster analyses suggested the existence of eight distinct populations (Fig. 5B), with basal cells dividing into two subpopulations based on the expression of *TP63* (BC1 with high *TP63* and BC2 with low *TP63*) and MCCs into four states (Fig. S3A). Amongst those clusters, *EHF* was expressed in all cells, with the highest levels in basal cells (Fig. 5C), as previously shown in primary cells. When comparing *EHF*^+/+^ and *EHF*^−/−^ cells, their distribution was similar in the MCC cluster but different in the basal cell cluster (Fig. 5D). Indeed, cluster BC1 was mainly made of *EHF*^+/+^ cells and expressed higher levels of *LRP1B*, *NCKAP5*, *SPARC* (osteonectin), *SPOCK1* or *CSMD3* while expressing lower levels of *SNTG1* or *S100A2* (Fig. S4B,C). Then, we explored lineage trajectory by pseudotime analyses with cycling basal cells as a starting point. Here, trajectories grew from cycling basal cells to MCCs with BC2 as an intermediate state for both *EHF*^+/+^ and *EHF*^−/−^ cells, confirming that the absence of EHF does not affect lineage specification for these cell types (Fig. S4D). Finally, we found 354 differentially expressed genes (DEGs) between *EHF*^+/+^ and *EHF*^−/−^ cells [|ln(FC)|>0.25 (FC, fold change) and Bonferroni-adjusted *P*-values <0.05]. Gene Ontology terms outlined keywords such as ‘cell differentiation’, ‘response to stress’ or ‘ion transport’ (Fig. 5E). Of particular interest, *ADGRL3*, *GOPC* and *PDE3* can affect CFTR activity (Nazarko et al., 2018; Cheng et al., 2005; Penmatsa et al., 2010); *CXCL17* and *SERPINB1* are linked to neutrophilic reaction (Choreño-Parra et al., 2020; Choi et al., 2019); and *ADAM28* was previously outlined by a study exploring the binding sites of EHF (Fossum et al., 2017). To confirm the strength of these findings, we explored the expression of a set of DEGs across several biological replicates by RTqPCR. Consistent with our scRNAseq results, we observed lower *EHF*, *RARB*, *NCKAP5* and *ADGRL3* mRNA expression, but higher *HLA-C*, *COL3A1*, *IGFBP4* and *PARM1* mRNA expression, in *EHF*^−/−^ compared to *EHF*^+/+^ clones (Fig. 5F). In conclusion, absence of EHF full-length protein affected the hiPSC-derived AEC transcriptional signature and, most specifically, basal cell states.

### The absence of EHF full-length protein improves CFTR function and epithelial integrity

**Fig. 6.**
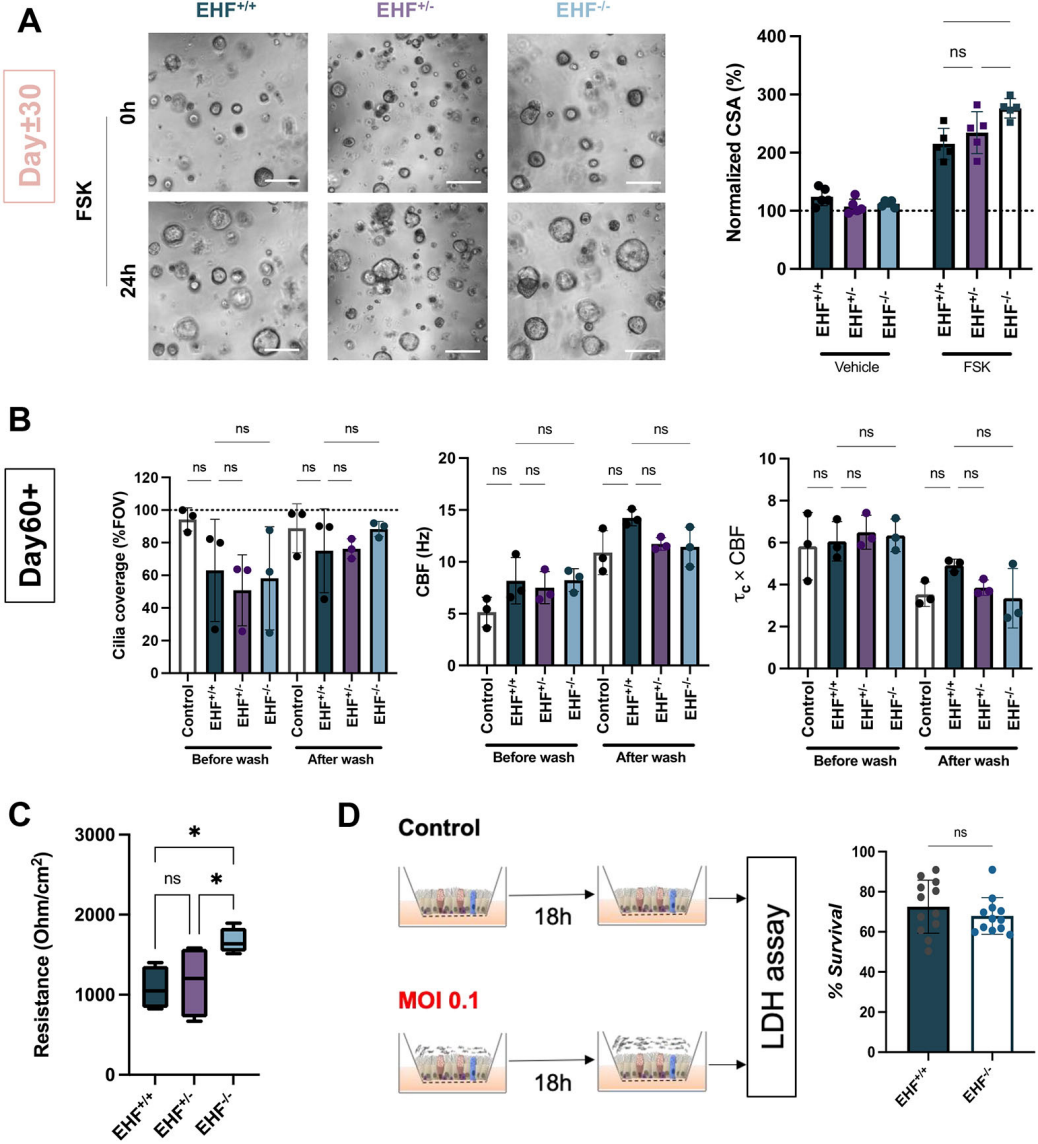
**Functional assays of EHF-edited clones.** (A) Swelling assay of Day 30 organoids treated by either vehicle (DMSO) or 10 μM forskolin (FSK) for 24 h. Representative images are shown on the left and quantification on the right. CSA, cross-sectional surface area. Reporter line, *n*=3 experimental replicates. Error bars represent s.d.; one-way ANOVA; ns, nonsignificant. (B) Percentage of field of view (FOV) covered by motile cilia (reporter line; left), cilia beating frequency (CBF; middle) and cilia beating synchronisation (τ_c_×CBF; right). (C) Transepithelial electrical resistance measured with a voltmeter. Reporter line, *n*=4 experimental replicates. Box and whisker plots represent quartiles, median, and minimum and maximum values. One-way ANOVA; ns, nonsignificant; *P<0.05. (D) Schematic of the experiment to determine survival of cells to 18 h of *P. aeruginosa* infection on Day 60 (left) and quantification of the results (right). Non-reporter line, *n*=3 experimental replicates, each in technical duplicates. Error bars represent s.d.; unpaired one-tailed Student's *t*-test; ns, nonsignificant. MOI, multiplicity of infection.

To further characterise the functional importance of EHF in AECs, we assayed key activities known to be impaired during pathological processes such as CFTR function, cilia motility, epithelial barrier integrity and response to bacterial infection. First, CFTR function was tested using forskolin-induced swelling (FIS) assay, a method widely used on primary cells and hiPSC-derived AEC organoids (Berical et al., 2022). All sublines showed swelling on organoids generated after 30 days of differentiation, as previously reported (Berical et al., 2022). Organoids treated with forskolin exhibited a larger cross-sectional surface area (CSA) than that of untreated controls, indicating that forskolin stimulated organoid growth. Notably, *EHF*^−/−^ organoids showed significantly greater swelling than *EHF*^+/−^ and *EHF*^+/+^ organoids (Fig. 6A). To confirm that this change was solely due to CFTR channel, we treated organoids with a CFTR inhibitor (CFTR-inh172). Here, CFTR-inh172 treatment significantly decreased CSA in both *EHF*^+/+^ and *EHF*^−/−^ organoids, suggesting that CFTR-inh172 prevented organoid swelling via CFTR and confirming that CFTR was the main channel involved in this finding (Fig. S4E). To better understand the underlying cause of this change, we performed RTqPCR analysis for *CFTR* expression. *CFTR* expression was not significantly different between *EHF*^+/+^ and *EHF*^−/−^ cells, suggesting the existence of alternative mechanisms (Fig. S4F). Second, cilia motility was assessed by microscopy on ALI cultures before and after PBS wash, which prompts cilia motility (Sone et al., 2021). Area covered by motile cilia, cilia beat frequency (CBF) and their coordination were unchanged (Fig. 6B). Third, to test epithelial integrity, TEER was analysed and found to be significantly increased in *EHF*^−/−^ compared to *EHF*^+/+^ AECs, suggesting potential differences in tight junctions (Fig. 6C; Fig. S4G). scRNAseq of ALI-cultured AECs outlined 33 DEGs termed as ‘cell adhesion’, and *CLDN7* and *CDH26* mRNA expression was found to be increased in *EHF*^−/−^ cells compared to *EHF*^+/+^ cells (Fig. S4H). However, RTqPCR validations showed that the expression of these genes was not statistically different, thereby suggesting that additional genes could be implicated in these TEER differences (Fig. S4I). Finally, we explored cell survival to bacterial infection, specifically cell resistance to *Pseudomonas aeruginosa*, a species of bacteria commonly responsible for chronic airway infections. There was no significant difference between *EHF*^−/−^ and *EHF*^+/+^ AECs in terms of viability post-infection (Fig. 6D). Overall, our data demonstrated that the absence of EHF protein increased CFTR function in organoids and TEER levels in ALI cultures, indicating multiple potential roles of EHF in the lung epithelium and potentially in lung airway physiopathology.

### hiPSC-derived AECs show a hypoxic response after 24 h at 1% O_2_

**Fig. 7.**
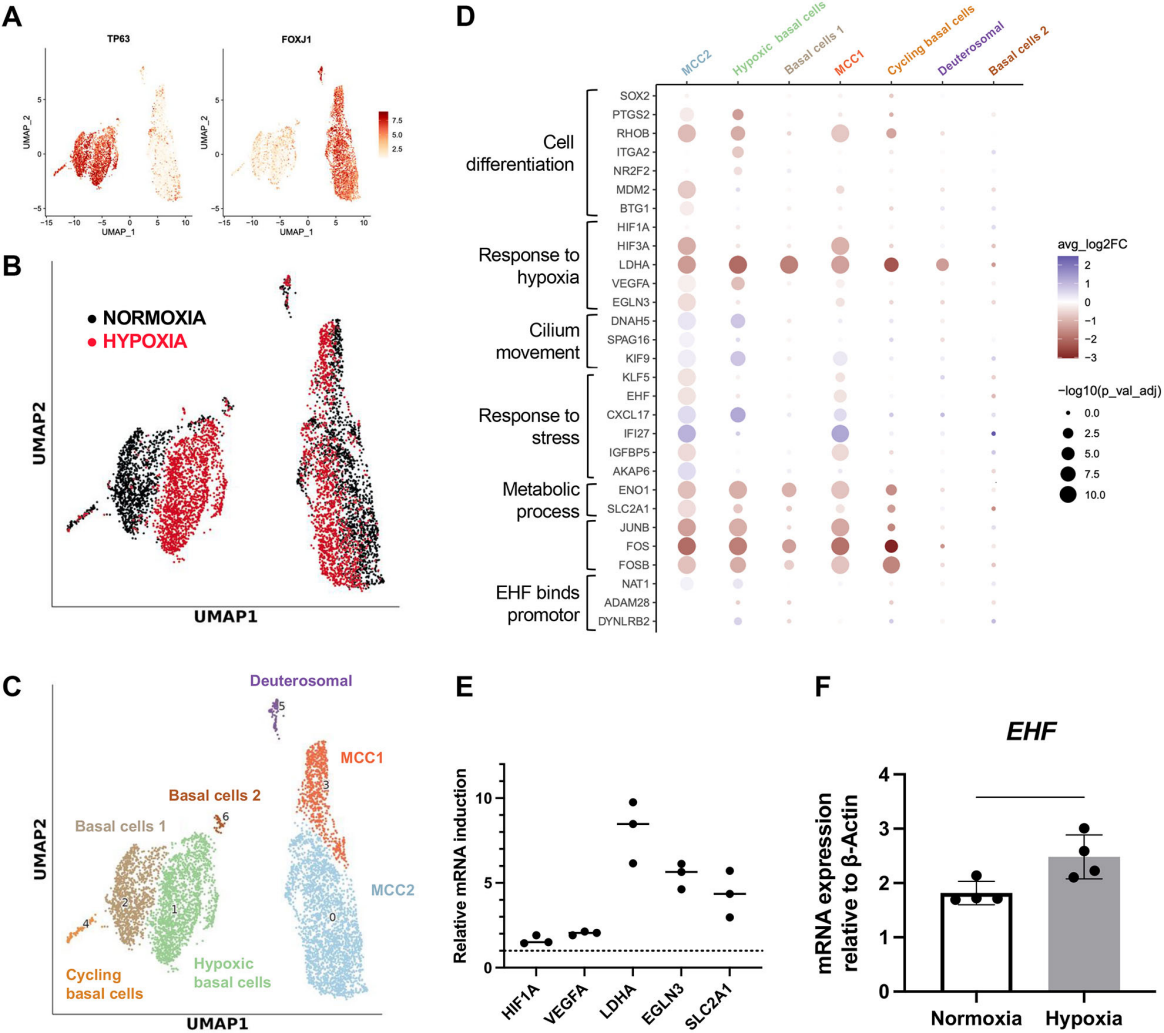
**Characterisation of the hypoxic response of our hiPSC-derived AECs.** (A) UMAP visualisation of cell expression for TP63 or FOXJ1. (B) UMAP visualisation of *EHF*^+/+^ cells in 21% (normoxia; black) or 1% (hypoxia; red) O_2_. (C) UMAP visualisation of cells coloured by cluster assignment at resolution 0.04. (D) Dot plots showing the differential expression of differentially expressed genes in the different clusters (resolution 0.04) in 21% (normoxia) compared to 1% (hypoxia) O_2_. Browns represent genes expressed more in hypoxia than in normoxia. (E) Relative expression of cells in hypoxia compared to cells in normoxia by RTqPCR of genes outlined by scRNAseq. (F) Gene expression by RTqPCR of *EHF* (reporter line) in 21% (normoxia) or 1% (hypoxia) O_2_ for 24 h, relative to housekeeping genes. *n*=3 experimental replicates. Error bars represent s.d.; unpaired two-tailed Student's *t*-test.

A major consequence of chronic lung diseases on the lung epithelium is hypoxia, driven by airway obstruction, mucus accumulation, structural remodelling and increased HIF-1α activation due to neutrophilic inflammation, all of which impair oxygen delivery to epithelial cells (Montgomery et al., 2017). Thus, we decided to investigate whether the absence of EHF could alter the response of AECs to hypoxia. We first validated that our *in vitro* model system could be used to study response to hypoxia. We opted for a short-term and intense hypoxic treatment (1% O_2_ for 24 h), which is known to induce a HIF-1α-specific response. Non-edited hiPSC-derived AECs were cultured in normoxia (21% O_2_) or in hypoxia (1% O_2_) for 24 h and then characterised by scRNAseq. Expression of *TP63* and *FOXJ1* confirmed the presence of basal cells and MCCs (Fig. 7A). Subpopulations expressing *MKI67* and *CDC20B* indicated the presence of cycling basal cells and deuterosomal cells, respectively (Fig. S3C). However, we did not observe the emergence of other cell types such as PNECs, previously reported to proliferate in hypoxia (Shivaraju et al., 2021). In the MCC cluster, hypoxic and normoxic cells clustered in proximity, whereas in the basal cell cluster they distinctively separated, indicating a significant change in their transcriptional signature (Fig. 7B). Subsequent clustering of these cells revealed six stable clusters (Fig. 7C; Fig. S3B). Notably, in basal cells, the levels of *ENO1*, *HIF1A* and *LDHA* were increased in hypoxia compared to normoxia, thereby confirming the importance of basal cells in the hypoxic response (Fig. S5A). Moreover, this significant response demonstrates the capability of cells generated in our model to effectively respond to variations in oxygen levels. To expand on our findings, we examined DEGs across all clusters and identified 978 genes with distinct expression patterns, which, upon term analysis, revealed their involvement in hypoxia (*HIF1A*, *HIF3A*, *ENOS*), stress response (*KLF5*, *EHF*, *CXCL17*, *IFI27*), metabolic processes (*ENO1*, *SCL2A1*) and cell death (*FOS*, *JUNB*) (Fig. 7D). Furthermore, in MCCs, genes implicated in cilia assembly and motility (*DNAH5*, *SPAG16*) were found to be downregulated in hypoxia, a feature previously outlined in primary cells (Zhang et al., 2022 preprint). To strengthen our results, we validated the induction of a set of upregulated genes by RTqPCR across different experimental and biological replicates. Accordingly, we found five key hypoxia-induced genes upregulated (*HIF1A*, *VEGFA*, *LDHA*, *EGLN3* and *SLC2A1*) (Fig. 7E). With regards to *EHF*, scRNAseq showed increased expression of *EHF* in hypoxia, suggesting involvement of *EHF* in hypoxic responses. To reinforce this observation, we conducted RTqPCR on multiple replicates, consistently showing a significant increase in *EHF* induction under hypoxia (Fig. 7F). Collectively, our data indicated that our hiPSC-derived AECs display a distinct hypoxic signature primarily driven by basal cells. Additionally, the induction of *EHF* in hypoxia suggests a potential role for this transcription factor in the hypoxic response.

### EHF loss downregulates the HIF-1α response in hypoxia

**Fig. 8.**
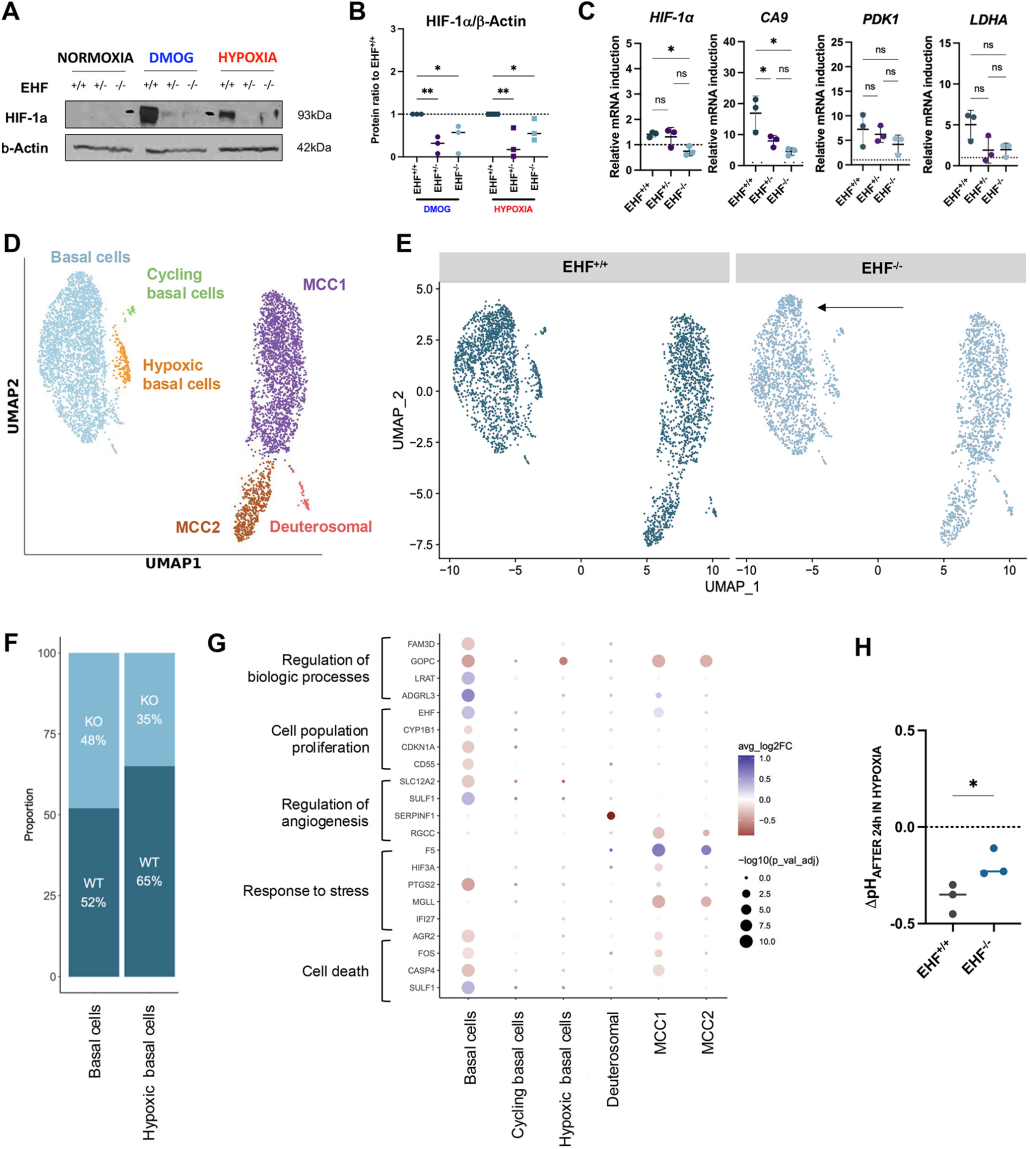
**Absence of EHF lowers the HIF-1α response, thus altering the epithelial extracellular pH.** (A) Representative western blot of HIF-1α in normoxia or after 24 h of treatment by prolyl hydroxylase inhibitor (DMOG) or 1% O_2_. β-actin was used as loading control. (B) Quantification of HIF-1α/β-actin per clones (reporter line). *n*=3 experimental replicates. Error bars represent s.d.; paired, one-way ANOVA; *P<0.05, **P<0.005. (C) Gene expression induction by RTqPCR of Day 60 cells (reporter line) after incubation in 1% O_2_ for 24 h. *n*=3 experimental replicates. Error bars represent s.d.; paired, one-way ANOVA; ns, not significant; *P<0.05. (D) UMAP visualisation of cells from additional lines coloured by cluster assignment. (E) UMAP visualisation of *EHF*^+/+^ and *EHF*^−/−^ cells. (F) Proportions of basal cells (cluster basal cells or hypoxic basal cells) in *EHF*^+/+^ or *EHF*^−/−^ cells. (G) Dot plots of differentially expressed genes in *EHF*^+/+^ compared to *EHF*^−/−^ cells in 1% O_2_ (hypoxia). Blues represent genes expressed more in *EHF*^+/+^ cells than in *EHF*^−/−^ cells. (H) pH variation of ALI cultures after 24 h in hypoxia (reporter line). *n*=3 experimental replicates. Error bars represent s.d.; paired, one-way ANOVA; *P<0.05.

Having validated that hiPSC-derived AECs can emulate hypoxic responses, we then investigated the significance of EHF in this pathological process. First, we assessed the expression of HIF-1α in *EHF*^−/−^ AECs after changes in oxygen levels and chemical induction by prolyl hydroxylase inhibitor (DMOG) (Dengler et al., 2014). The levels of HIF-1α were significantly decreased in *EHF*^+/−^ and *EHF*^−/−^ cells at protein and mRNA levels in the reporter cell line, but the decrease in HIF-1α was not statistically significant in the non-reporter hiPSC line (Fig. 8A-C; Fig. S5B-D). This difference may be attributed to biological differences between both hiPSC lines, such as variations in gene expression or compensatory pathways. Then, we explored HIF-1α-induced gene expression by RTqPCR. Interestingly, we observed a statistical decrease in the expression of *CA9* (Fig. 8C; Fig. S5D). For deeper transcriptional analysis, we then performed scRNAseq. As expected, clustering showed six clusters, indicating that the absence of EHF does not affect the cellular diversity of hiPSC-derived AECs in hypoxia (Fig. 8D; Fig. S3C). Notably, *CA9* was mostly expressed by hypoxic basal cells (Fig. S5E). Moreover, this cluster was constituted of 65% *EHF*^+/+^ cells and 35% *EHF*^−/−^ cells (Fig. 8F). In examining the overall distribution of *EHF*^+/+^ and *EHF*^−/−^ cells, minimal changes were noted, except for the emergence of a subpopulation within *EHF*^−/−^ basal cells (black arrow, Fig. 8E). Importantly, this subpopulation expressed high levels of *ADAM28* (Fig. S5E), the promoter of which was previously shown to be bound by EHF (Fossum et al., 2017). Next, we examined DEGs between *EHF*^+/+^ and *EHF*^−/−^ cells in hypoxia and identified 289 genes for which corresponding term analysis included stress response (*F5*, *PTGS2*), hypoxia (*HIF3A*, in particular), regulation to angiogenesis (*SULF1*, *SERPINF1*) and cell death (FOX, *CASP4*) (Fig. 8G). Lastly, we aimed to understand the functional impact of these variations, by assessing cell survival and pH homeostasis, both reliant on HIF-1α. Cell survival in 1% oxygen, tested by viability assay, was not impacted by the loss of EHF protein (Fig. S5E). However, the absence of EHF significantly decreased the hypoxic-driven acidification of our ALI cultures (Fig. 8H). Altogether, these data propose a non-canonical mechanism for HIF-1α regulation by EHF, which could control the impact of hypoxia on lung epithelium.

## DISCUSSION

In this study, we used hiPSC-derived AECs to investigate the function of *EHF* in the lung airway epithelium, specifically during disease-related processes. First, we validated our *in vitro* model by showing the presence of AECs, including lung stem cells, basal cells, MCCs, deuterosomal cells and SCGB3A2-expressing secretory cells. Notably ALI-cultured AECs, comprising basal cells, MCCs and deuterosomal cells, express EHF at similar levels to primary cells. Then, we used CRISPR/Cas9 gene editing to derive hiPSC lines that can be used to produce AECs devoid of EHF full-length protein. scRNAseq analysis revealed a set of DEGs in AECs grown in ALI cultures and, notably, variations in basal cell states. Furthermore, the loss of EHF protein led to enhanced CFTR function, significantly increased TEER and significantly reduced HIF-1α-driven hypoxic response, causing alterations in epithelial pH. Thus, these data showed that EHF plays an important role in lung epithelium grown *in vitro*.

Collectively, our results demonstrated the potential of our hiPSC-derived AEC platform for functional exploration within the context of lung airway pathological processes. Our protocol allowed the production of lung stem cells, SCGB3A2-expressing secretory cells, basal cells, deuterosomal cells and MCCs in our organoids or ALI cultures. Furthermore, generated MCCs display characteristics of mature cells, exhibiting coordinated cilia movement, a feature never quantified before in hiPSC-derived MCCs, and impaired ciliogenesis in hypoxia, showing that we were able to reproduce physiological and pathological processes previously described in primary cell models (Jiao et al., 2022). Nevertheless, several limitations need to be considered. Importantly, our culture system did not contain mature secretory cells or rare cell types such as ionocytes, tuft cells or PNECs. Thus, our platform does not allow for the study of these cell types or complex cellular interplays. To our knowledge, there are no existing hiPSC-derived models capable of generating all these specified cell types. However, new protocols show the emergence of rarer cell types such as ionocytes (Wang et al., 2023). In the future, adding IL13 or IL1β to our ALI cultures could promote the generation of mucosecretory cells, and further sorting steps could enhance the emergence of rarer cell types, both these approaches contributing to developing a more diverse system closer to primary tissue (Chen et al., 2019).

Importantly, our findings are consistent with those of previous studies. Of particular interest, Fossum et al. (2014, 2017) have investigated the transcriptional network of EHF in Calu-3 cells. There, chromatin immunoprecipitation with sequencing data identified binding sites for EHF in loci relevant to genes referenced by our study, including *ITGA2*, *CXCL17*, *HIF1A*, *ROBO1*, *RARB*, *PDE3A* and *ADGRL3*. The roles of these genes include development (*RARB*), fibrotic processes (*ROBO1*), immune response (*CXCL17*), CFTR regulation (*PDE3A*, *ADGRL3*) and hypoxic response (*HIF1A*, *PHD2* and *PHD3*). This study also demonstrated that EHF binds to the promoter of ADAM28. Under hypoxic conditions, a population of *EHF*^−/−^ cells expressing high levels of ADAM28 was observed, which was absent in *EHF*^+/+^ cells. These findings suggest that EHF represses ADAM28 expression in hypoxia through transcriptional regulation. As ADAM28 is important for viral and immune system responses, lower levels of *EHF* could be beneficial to infectious and/or inflammatory pathological processes (Baurakiades et al., 2014; de Seabra Rodrigues Dias et al., 2022). As for functional assays, similarly to the aforementioned study on Calu-3 cells (Fossum et al., 2017), we found TEER values to be significantly increased in *EHF*^−/−^ clones, thereby suggesting a role for the transcription factor in controlling tight junctions. Accordingly, we observed increased expression of *CLDN7* in *EHF*^−/−^ cells. This claudin promotes paracellular permeability of chloride ions, and thus higher expression of this gene could influence aspects of diseases in which ion channels are implicated, such as CF (Tatum et al., 2007).

Knockdown of EHF was previously shown to increase *CFTR* expression in Calu-3 cells and HBECs via *cis*-regulatory upstream elements (Mutolo et al., 2018). Our study reinforces these findings as our organoid swelling assays showed enhanced CFTR function in *EHF*^−/−^ cells. However, we did not see a significant alteration in *CFTR* mRNA levels, although *CFTR* mRNA levels were increased in *EHF*^−/−^ cells. Inter-replicate variability and/or a more complex role of *EHF* in CFTR protein function, beyond sole transcriptional repression, may explain the lack of statistical significance. Indeed, the expression of multiple CFTR-associated genes was altered in *EHF*^−/−^ cells, such as the cAMP inhibitor *ADGRL3* or the cAMP reducer *PDE3A* (Nazarko et al., 2018; Penmatsa et al., 2010). Our results indicate that reduced *EHF* expression enhances CFTR function, suggesting that lower levels of EHF could be beneficial in CF. Further studies will help clarify how EHF expression levels impact cells in individuals with CF. For instance, significant insights could be provided by comparing the swelling in *EHF*^−/−^ organoids with that in *EHF*^+/+^ organoids treated with CF modulators such as Kaftrio/Trikafta. If the swelling of *EHF*^−/−^ organoids matches that of *EHF*^+/+^ organoids treated with Kaftrio, it would highlight the role of EHF in modulating cellular responses, warranting further investigation into the interplay between these treatments and genetic modifications.

The most significant finding of our study is the role of EHF in hypoxia. Intraluminal airway muco-obstruction causes airway epithelial hypoxia in a broad range of chronic lung diseases such as COPD, CF or COVID-19. Recently, Mikami et al. (2023) confirmed the presence of a clear hypoxic response in the airway epithelial tissues of individuals with CF, non-CF bronchiectasis, primary ciliary dyskinesia and COVID-19, evidenced notably through the induction of EGLN3 or EGLN1 (Mikami et al., 2023), thus confirming the relevance of hypoxia as a prime pathological process for multiple lung diseases. Here, we found that the absence of EHF functional protein reduced HIF-1α levels in cells cultured under hypoxic conditions, resulting in a decrease in CA9, which in turn limited epithelial acidification. As acidity is known to increase mucus viscosity, EHF is likely to impact mucociliary clearance through this process (Tang et al., 2016). Therefore, CA9 inhibitors, currently used in oncologic indications, could be investigated as a promising therapeutic avenue (Kalinin et al., 2021). In addition to pH changes, recent publications showed that HIF-1α induces profibrotic and proinflammatory phenotypes in the lung, which are detrimental in the context of lung airway diseases (Brereton et al., 2022; Epstein Shochet et al., 2021). As for the mechanism(s) involved, we found that the loss of EHF reduced *HIF1A* transcription, but we cannot rule out additional and/or compensatory mechanisms such as a role in HIF-1α degradation, for instance. Thus, the role of EHF in hypoxia may have broader implications, and our research paves the way for further studies in the context of hypoxia.

In conclusion, our study uncovers diverse functions for EHF in the airway epithelium. These functions suggest that a decrease in EHF could have beneficial implications in chronic lung diseases. Thus, new therapies controlling EHF expression could help to decrease disease severity.

## MATERIALS AND METHODS

### hiPSC line generation and culture

hiPSCs used for this study are a non-reporter line derived in our laboratory (FS13B) (Kilpinen et al., 2017) and a reporter line [CFF-iPSC-NKX2.1(GFP)-TP63(mCherry) WT/WT] kindly provided by the Cystic Fibrosis Foundation. Cells were cultured on 10 µg/ml vitronectin (StemCell Technologies)-coated plates in hiPSC basal medium (Table S1) supplemented with 25 ng/ml FGF2 (Cambridge University) and 2 ng/ml TGFb1 (Bio-Techne). Medium was renewed daily. Cells were passaged using 0.5 mM EDTA (Life Technologies) for 5 min at room temperature (RT).

### Differentiation of hiPSCs into AECs

hiPSCs were dissociated using Accutase^®^ (Invitrogen) at 37°C, counted and seeded onto gelatin (Sigma-Aldrich)-coated 12-well plates in hiPSC medium supplemented with 10 μM Y-27632 (Selleck). Twenty-four hours after seeding, medium was renewed with hiPSC basal medium without Y-27632 (Day 0). Definitive endoderm differentiation was induced following a protocol previously published by our group (Hannan et al., 2013). Endoderm differentiation was induced for 3 days using Endoderm medium#1 at Day 1 and Day 2 (Table S1) and Endoderm medium#2 at Day 3 (Table S1). Medium was supplemented with 100 ng/ml Activin A (Cambridge University), 80 ng/ml FGF2 (Cambridge University), 10 ng/ml BMP4 (R&D Systems), 3 μM CHIR99021 (Tocris) and 10 μM LY294002 (Promega) at Day 1; Activin A, FGF2, BMP4 and LY294002 at Day 2; and Activin A and FGF2 at Day 3. At Day 4, the medium was changed to lung base medium (LBM; Table S1). From Day 4 to Day 8, LBM was complemented with 100 ng/ml noggin (R&D Systems) and 10 μM SB431542 (Tocris), and from Day 9 to Day 16 with 3 μM CHIR99021, 10 ng/ml BMP4 and 50 nM retinoic acid (Sigma-Aldrich). The medium was renewed every day.

### Enrichment of *NKX2.1*^+^ lung progenitors by fluorescence-activated cell sorting (FACS)

At Day 16, cells were dissociated with TrypLE™ Express (Thermo Fisher Scientific). The cell suspension was then filtered, counted and stained for 30 min at 4°C with mouse anti-CPM antibody (Fujifilm) and secondary anti-mouse Alexa Fluor 488 (Life Technologies) for the non-reporter line. After washing, CPM^HI^ or NKX2.1^HI^ cells were sorted using a BD Influx (BD Biosciences). An unstained population was used for gating.

### Culture, cell sorting for purification and maintenance of iPSC-derived basal cells

CPM^HI^ or NKX2.1^HI^ cells were re-plated in growth factor-reduced Matrigel (Corning) domes and cultured in LBM supplemented with 250 ng/ml FGF2 (R&D Systems), 100 ng/ml FGF10, 50 nM dexamethasone (R&D Systems), 100 nM 8-bromoadenosine 3′,5′-cyclic monophosphate sodium salt (cAMP; Sigma-Aldrich), 100 μM 3-isobutyl-1-methylxanthine (IBMX; Millipore-Sigma) and 10 μM Y-27632. Medium was replaced every other day until Day 30. At Day 30, organoids were either split for the non-reporter line and re-seeded in new domes or sorted for the reporter line. For sorting, domes were dissociated using TrypLE™ Express, and double-positive NKX2.1^+^/TP63^+^ cells were seeded into fresh Matrigel domes. Cells were then cultured with Pneumacult-Ex Plus (StemCell Technologies) supplemented with 1 μM A83-01 (Tocris), 1 μM DMH1 (Tocris) and 10 μM Y-27632. At Day 40, organoids were dissociated using the same technique, stained for anti-NGFR-BV421 (BioLegend), and sorted for NKX2.1^+^/TP63^+^/NGFR^+^ for the reporter line or NGFR^+^ only for the non-reporter line. Subsequent cells were seeded onto transwell inserts for culture and placed in similar conditions. For ALI cultures, sorted cells were seeded onto PET transwells (Corning, 3460), coated with 115 µg/ml Matrigel, the top chamber was lifted, and medium used was Pneumacult ALI (StemCell Technologies) and changed every other day for 3-4 weeks.

### hiPSC genetic editing

EHF was targeted from hiPSCs as previously described (Tilson et al., 2021). Briefly, two single gRNAs were used to target Exon#3 of *EHF* in hiPSCs, and Cas9 protein was used to allow targeting. gRNAs were all purchased from Synthego^®^, and details on their sequences can be found in Table S2. Cas9 was kindly provided by the Department of Biochemistry of the University of Cambridge. Cells were edited using a Lonza nucleofection kit, and subsequent pools were cloned. DNA from selected clonal colonies was extracted, amplified and purified before sequencing. To determine knockout efficiency, we genotyped our clones using a platform service (Genewiz^®^). Corresponding sequences were uploaded to two software tools: Inference of CRISPR Edits (ICE) and Tracking of Indels by Decomposition (TIDE). Cells with sought genotypes were then amplified and differentiated to explore *EHF* expression at mRNA and protein levels by RTqPCR and western blotting, respectively.

### Hypoxic chamber treatment, survival assay and pH measurement

Day 60 cells were cultured in a Whitley H35 Hypoxystation (Don Whitley Scientific) at 37°C for 24 h at 5% CO_2_, 1% O_2_ and 94% N_2_. After 24 h, cells were harvested, counted or stained depending on the assay performed. Cell viability was determined by incubating cells with 1:10 Presto Blue reagent (Invitrogen) in Pneumacult ALI medium at 37°C for 4 h. The EnVision plate reader (excitation/emission of 560 nm/590 nm) was used to measure the corresponding fluorescence. For pH measurement, bottom-well media were collected before and after 24 h incubation, and pH values were measured using a pH meter (Jenway).

### RNA extraction, cDNA synthesis and RTqPCR

RNA was extracted using a GenElute RNA-extraction kit (Sigma-Aldrich) according to the manufacturer's instructions. Purified RNA was eluted in 30 μl nuclease-free water (Life Technologies), and its concentration was measured using a Nanodrop spectrophotometer (Thermo Fisher Scientific). Then, 500 ng RNA was mixed with 0.5 μl random primers (Promega), 1 μl dNTPs (Promega), topped up to 11.8 μl with nuclease-free water (Sigma-Aldrich) and incubated at 65°C for 5 min to denaturate the RNA. Next, 4 μl first-strand buffer (Thermo Fisher Scientific), 2 μl dithiothreitol (Thermo Fisher Scientific), 0.5 μl RNase OUT (Life Technologies) and 0.2 μl Superscript II (Thermo Fisher Scientific) were added to each reaction. The resulting cDNA was diluted with nuclease-free water to a total volume of 600 μl. Each RTqPCR reaction was performed with 3 μl cDNA, 5 μl KAPA SYBR Fast (Sigma-Aldrich), 0.4 μl of 5 μM forward primer and 0.4 μl of 5 μM reverse primer. See Table S2 for primer sequences. All primer pairs were validated to ensure only one product and a PCR efficiency of 100% (±10%). RTqPCR was performed using a QuantStudio™ 5 Real-Time PCR System (Thermo Fisher Scientific). mRNA expression was analysed using the ΔΔCt method normalising the results to an average of three internal reference genes (housekeeping genes): *UBC*, *RPLPO* and *PBDG* in normoxia or β-actin in hypoxia. Primer sequences are summarised in Table S2.

### Immunofluorescence staining

Samples were washed with PBS (Thermo Fisher Scientific), fixed at RT for 20 min with 4% paraformaldehyde (Alfa Aesar), washed and blocked for 1 h with 10% donkey serum (Bio-Rad) supplemented with 0.1% Triton X-100 (Sigma-Aldrich) in PBS at RT. Primary antibodies were used to stain samples overnight at 4°C in 1% donkey serum supplemented with 0.1% Triton X-100 in PBS. After PBS washes, secondary antibodies were used to stain samples overnight at 4°C. All antibodies used are listed in Table S3. Samples were washed and stained with DAPI for 10 min at RT and then washed again. Stained samples were imaged using a Zeiss LSM 710 or 910 confocal microscope. Images were analysed using FIJI. The antibodies used and their concentrations can be found in Table S3. For the staining of ALI inserts, each transwell membrane was cut into four pieces and separately stained in wells of a 48-well plate. After staining and washing, inserts were mounted on glass. For organoid staining, PBS washes were extended to 45 min.

### Western blotting

Cells were detached and washed in PBS. Then, the cell pellet was resuspended in ice-cold RIPA buffer (150 mM NaCl, 50 mM Tris-HCl, pH 8.0, 1% NP-40, 0.5% sodium deoxycholate, 0.1% sodium dodecyl sulfate) supplemented with protease and phosphatase inhibitors for 10 min. Proteins were quantified by BCA assay (Pierce) following the manufacturer's instructions using a standard curve generated from bovine serum albumin and read (600 nm) on an EnVision 2104 plate reader. After dosage, 4× NuPAGE LDS sample buffer (Life Technologies) plus 1% β-mercaptoethanol was added, and samples were heated at 90°C for 5 min. Then, 40-60 μg of protein per sample was run on a 4-12% NuPAGE Bis-Tris Gel (Life Technologies) and transferred to a PVDF membrane by liquid transfer using NuPAGE Transfer buffer (Life Technologies, NP0006). Then, membranes were blocked at room temperature for 1 h in PBS 0.05% Tween 20 (PBST) supplemented with 4% non-fat dried milk and stained overnight at 4°C with primary antibodies. After three washes in PBST, membranes were incubated for 1 h at RT with horseradish peroxidase (HRP)-conjugated secondary antibodies diluted in blocking buffer, then washed a further three times before being incubated with Pierce ECL2 Western Blotting Substrate or SuperSignal™ West Pico PLUS Chemiluminescent Substrate (Thermo Fisher Scientific) and, finally, exposed to X-ray film. Antibodies used are listed in Table S3.

### Statistical methods

Data were processed using GraphPad Prism software (Version 9) for both visualisation and statistical analysis for RTqPCR or flow cytometry data. Unpaired, two-tailed Student's *t*-tests were used for comparisons involving only two groups, and one-way ANOVA was used when considering multiple groups.

### scRNAseq

Quality checks, alignment and feature quantification were performed using 10x Cell Ranger v6.1.1, on the GRCh38 reference transcriptome (Fig. S6). The distributions of per-cell sequencing depths, number of features, and proportions of reads incident to mitochondrial (MT) and ribosomal protein-coding (RP) genes, per cell, were summarised using violin plots. Using saturation plots, we confirmed the logarithmic relationship between the number of detected features per cell and the nCount [number of unique molecular identifiers (UMIs) per cell], which suggests that sequencing saturation was not reached. Based on these summaries, we excluded cells with <2500 unique features, <5000 or >75,000 UMIs, and >20% MT or >25% RP genes; the filtering reduced the number of cells from 15,163 to 11,534. After filtering, MT and RP genes were excluded from the count matrices and, subsequently, SCTransform normalisation was applied (Hafemeister and Satija, 2019). Further quality checks were based on principal component analysis (PCA) and UMAP summaries; raw and normalised nCounts, MT% and RP% were represented on a colour gradient, to assess technical variation; we saw several regions on the UMAP with anti-correlated %MT and %RP, i.e. higher %MT/lower %RP or vice versa; based on manual curation of expressed genes, we concluded that these regions correspond to different cell types so no further correction or filtering was applied. The cell cycle was computationally inferred using the CellCycleScoring function in Seurat, on the annotated list of cell cycle genes. The Pearson residuals of the 2500 highly variable genes were used for PCA, and the first 30 principal components (PCs) were used to calculate the UMAP (Becht et al., 2019); the stability of the selected genes was assessed using ClustAssess (Shahsavari et al., 2022 preprint). A similar assessment was performed separately for only normoxia, only hypoxia and only wild-type samples; the same gene selection was deemed appropriate in each case. Cells were clustered with Seurat v4.2.1 using the SLM algorithm; the clustering was applied separately on the normoxia, hypoxia and wild-type samples, based on a 30 (normoxia and wild type) and 25 (hypoxia) shared nearest neighbour graph. The number of neighbours in the graph, the robustness of the clustering algorithm and the resolution were selected using ClustAssess; stable resolutions were 0.1 for normoxia, 0.05 for hypoxia and 0.04 for wild type. Marker genes for each cluster were identified using the Wilcoxon rank sum test in Seurat; genes with |ln(FC)|>1 or |ln(FC)|>0.25 and Bonferroni-adjusted *P*-values <0.05 were selected and used to manually curate cluster labels. The same parameters were used to find DEGs comparing wild-type and knockout cells (in normoxia and hypoxia) and normoxia and hypoxia cells (wild type). The same analysis was performed specifically for normoxia basal cells (clusters 0, 4, 5) and hypoxia basal cells (clusters 0, 3, 5). Gene set enrichment analysis was performed with g:profiler (Raudvere et al., 2019), using as selected subset the DEGs [|ln(FC)|>0.25 and Bonferroni-adjusted *P*-values <0.05] between wild type/knockout and normoxia/hypoxia, overall and per cluster, and as background set all genes returned by SCTransform across all samples and clusters. Enrichment terms with false discovery rate-adjusted *P*-values of <0.05 were further explored. Pseudotime trajectories were calculated using Monocle3 v1.3.1 with default parameters on the original UMAP representation (Qiu et al., 2017; Cao et al., 2019; Trapnell et al., 2014). The starting point was chosen as the cells expressing (>0) *TP63*, *TOP2A*, *MKI67* and *KRT5* and not expressing (=0) *FOXJ1* and *SNTN*.

### Cilia motility by microscopy

High-speed videos of the ciliated epithelia at the ALI stage were collected as described before (Fradique et al., 2023). Twenty arbitrary fields of view (FOVs) for each sample were imaged before and after mucus removal. The FOVs were pre-selected equally spaced to include information both in the middle and on the edge of the tissue, aiming to avoid any potential selection bias. Mucus wash was performed by incubating the epithelial apical surface for 20 min in 200 μl PBS, followed by removal of the solution. All samples were kept at 37°C, 100% humidity, 5% CO_2_ and in ALI media throughout the imaging. High-speed videos of commercially available human bronchial epithelia reconstituted *in vitro* (MucilAir™, Epithelix) were analysed and used to compare MCCs properties of hiPSC-derived AECs with those of primary AECs-derived epithelia. Three inserts from three different healthy donors were purchased (Epithelix Sàrl), maintained following the protocol provided by the company and imaged. Properties of cilia dynamics (CBF and motile cilia coverage) and ciliary coordination were quantified using two methods developed in the Cicuta laboratory based on fast Fourier transform (FFT). Twenty videos for each sample were analysed for the measurement of CBF and coverage using the MATLAB code. The algorithm is based on correlation properties of pixel intensities over time and relies, in the first place, on differences in the autocorrelation function in areas with and without beating cilia, performed in boxes of different sizes within the FOV, to determine the regions in which movement is present. This is then followed by a Fourier power spectrum analysis to determine the distribution and mean CBF only of the areas covered in motile cilia. The ten most covered FOVs for each sample before and after mucus removal were analysed with the multi-differential dynamic microscopy (DDM) algorithm. Briefly, the basic DDM analysis, based on FFT of image differences over time and used to evaluate dynamics in optical microscopy videos, is run in a progressively smaller region within the FOV, thus extracting the image structure function (ISF) for each box size. The ISF at fixed box size (fixed spatial frequency *q*) when measured on videos of cells with motile cilia consists of a signal that is approximately a damped oscillation as a function of the time interval. Fitting the ISF as a damped oscillator allows us to measure the decay rate of its envelope, which indicates the time taken for that region to lose coherence. Therefore, the decay time τ_c_ provides information about how coordinated the cilia beating is (how much ciliary beating is in phase within a certain region). Here, we decided to quantify the coordination in boxes of size comparable to that of a cell (∼10 μm side) in the nondimensional parameter τ_c_×CBF. Note that the two quantities are evaluated as the average of the characteristic frequency and decay time of all the boxes of that size in which movement is detected in the FOV. At 60× magnification, 1 px=0.098 μm. The inverse pixel size *k*=10.2 μm^−1^ and the box size *L*=96 px define the spatial frequency *q*=2π*k*/*L*=0.67 μm^−1^.

### CFTR function by FIS assay

The method used for FIS assay was performed as described in Berical et al. (2022). Briefly, Day 30 cells were seeded as organoids and pictures were taken at 0 h. Cells were then treated with either vehicle (DMSO), CFTR activator (10 μM forskolin), or CFTR activator (10 μM forskolin) supplemented with CFTR inhibitor (CFTR-inh172) for 24 h. After 24 h, repeat images were captured and analysed identically to the pre-forskolin images above. Saved images (.tif) were analysed using Fiji. A minimum of 30 organoids were analysed per well, and technical duplicates were performed for each experimental replicate. Individual sphere CSA was measured at each time point and calculated as a percentage of initial CSA: (surface organoid at 0 h/surface organoid at 24 h)×100.

### Bacterial infection and cell survival analysis

hiPSC-derived AECs were infected for 1 h at a multiplicity of infection (MOI) of 0.1:1 with the wild-type strain of *Pseudomonas aeruginosa* PAO1 (ATCC, BAA-47-B1) and assessed for survival at specific times post-infection. Briefly, PAO1 overnight culture was spun down at 1000 ***g*** for 10 min, washed once in PBS, and resuspended in cell medium at the adjusted MOI. hiPSC-derived AECs were washed in warm PBS for 10 min and submerged for 1 h in medium containing the adjusted bacterial load. After 1 h, the infection medium was removed, and cells were placed back at 37°C and 5% CO_2_ under ALI conditions. At specific time points, hiPSC-derived AECs were washed with PBS and then lysed to assess survival using a CyQuant LDH cytotoxicity assay (Thermo Fisher Scientific) following the manufacturer's instructions. Data analysis was made based on LDH retained, i.e. the percentage of cell survival compared to the control.

## Supplementary Material

10.1242/dmm.052106_sup1 Supplementary information
