# Supplementary material for: An in vitro model of the epithelial airway reveals a key function for EHF in lung homeostasis and disease
Source: Dis Model Mech. 2025 Jul 1;18(6):dmm052106. doi: 10.1242/dmm.052106 (PMC12264733; doi:10.1242/dmm.052106)
Supplement: Supplementary information [file dmm-18-052106-s1.pdf]

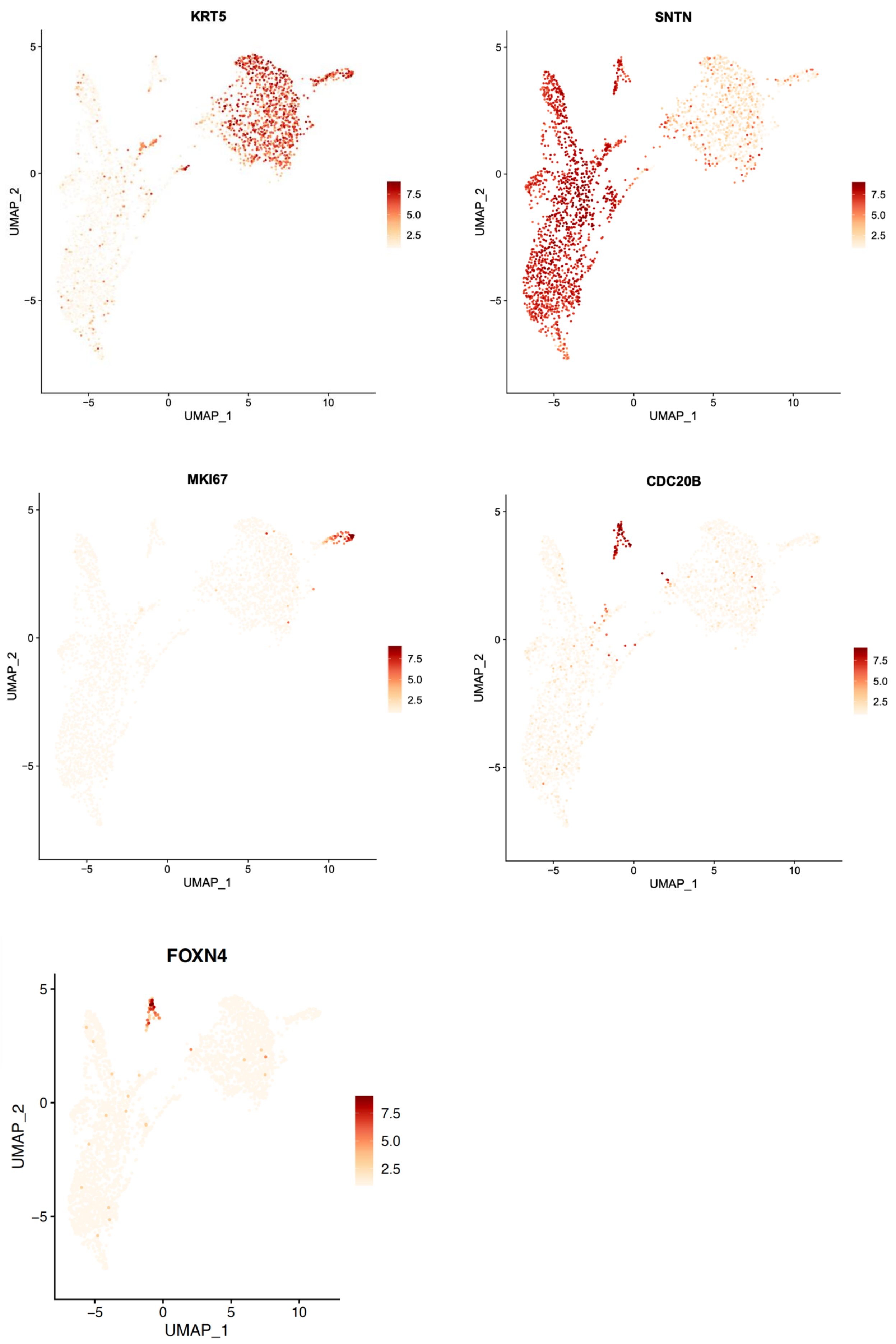

Fig. S1. Further characterization of our model.

UMAP visualisations of expression for KRT5, SNTN, MKI67, CDC20B, and FOXN4 in Day60 ALI culture cells.

Disease Models & Mechanisms • Supplementary information

A

Clone	Sequence of EHF protein predicted
Wild-type	MILEGGGVMNLNPGNNLLHQPPAWTDSYSTCNVSSGFFGGQWHEIHPQYWTKYQV WEWLQHLLDTNQLDANCIPFQEFDINGEHLCSMSLQEFTRAAGTAGQLLYSNLQHLK WNGQCSSDLFQSTHNVIVKTEQTEPSIMNTWKDENYLYDTNYGSTVDLLDSKTFCRA QISMTTTSHLPVAESPDMKKEQDPPAKCHTKKHNPRGTHLWEFIRDILLNPDKNPGLIK WEDRSEGVFRFLKSEAVAQLWGKKKNNSSMTYEKLSRAMRYYYKREILERVDGRRL VYKFGKNARGWRENEN (35kDa)
Clone EHF$^{-/-}$ of reporter iPSC line edited with gRNA#1	MILEGGGVMNLNPGNNLLHQPPAWTDSYSTCNVSSGFFGGQWHEIHPQYWTKYQV VGVAPAPPGHQPAGCQLYPFPRVRHQRRAPLQHEFAGVHPGGRDGGAAPLQQLAA SAVER (12.48 kDa)
Clone EHF$^{-/-}$ of non-reporter iPSC Line edited with gRNA#2	MILEGGGVMNLNPGNNLLHQPPAWTDSYSTCNVSSGFFGGQWHEIHPQYWTRCGS GSSTSWTPTSWMPIVSLSKSSTSTASTXAA (8.99 kDa)
EHF polyclonal antibody immunogen sequence: MILEGGGVMN LNPGNNLLHQ PPAWTDSYST CNVSSGFFGG QWHEIHPQYW TKYQVWEWLQ HLLDTNQLDA NCIPFQEFDI NGEHL	

Color code: Binding domain of the EHF protein.
Last amino acid before changes from edition driven by gRNA#1.
Last amino acid before changes from edition driven by gRNA#2.

B

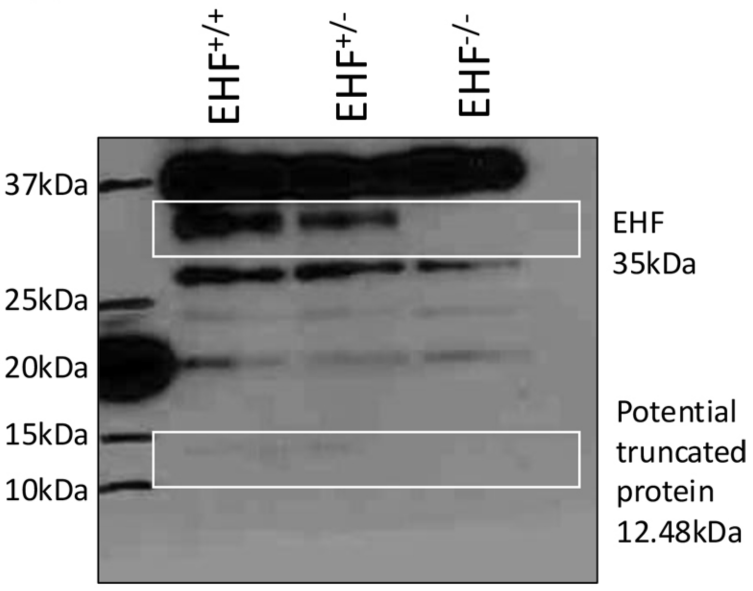

Fig. S2. Complementary information on EHF protein.

(A) Predicted protein sequence of different edited clones. **(B)** Representative WB of EHF in the reporter line.

Disease Models & Mechanisms • Supplementary information

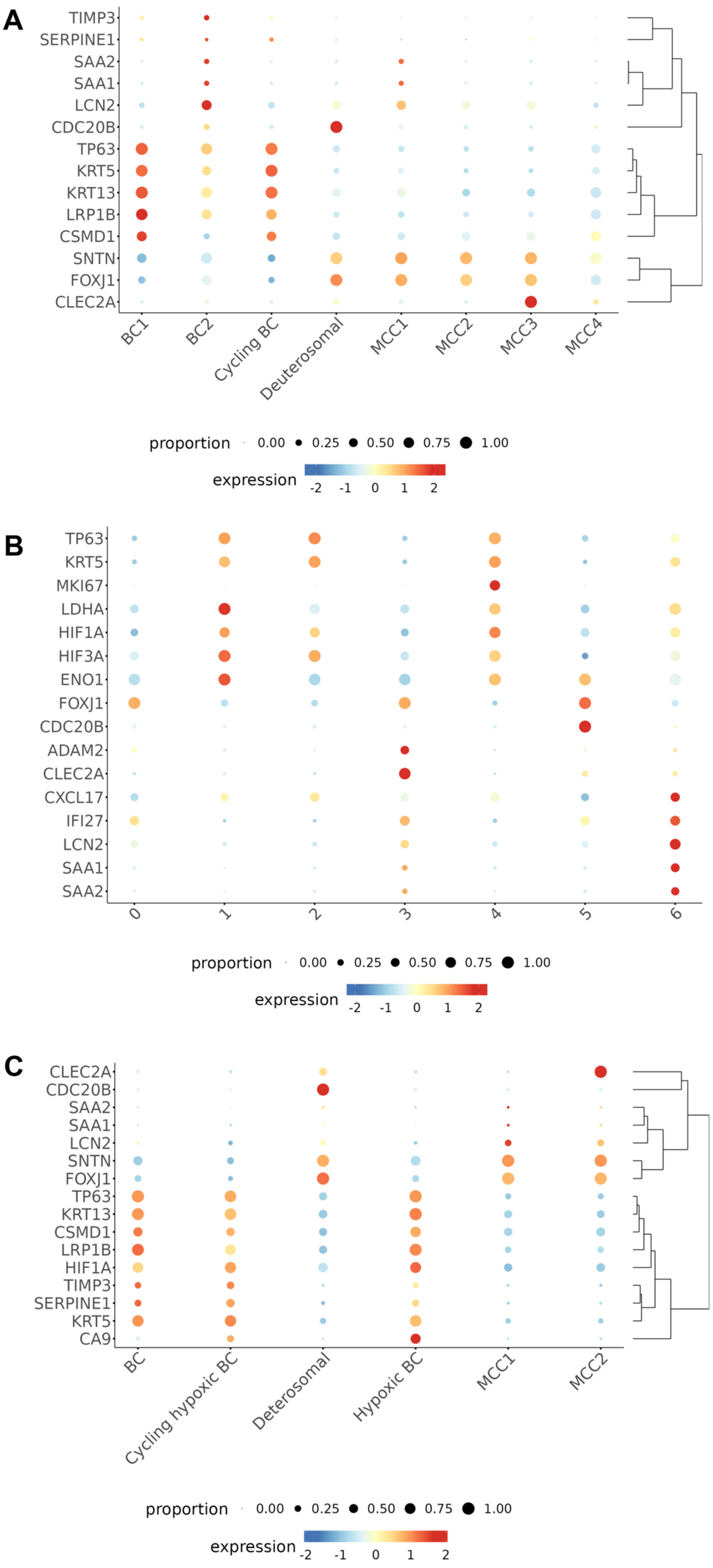

Fig. S3. DEGs involved in clustering.

(A&C) (A) Dot plot showing differentially expressed genes in EHF$^{+/+}$ and EHF$^{-/-}$ (reporter line) driving clustering in normoxia or **(C)** in hypoxia. **(B)** Dot plot analysis of differentially expressed genes driving cell clustering in normoxia and hypoxia.

Disease Models & Mechanisms • Supplementary information

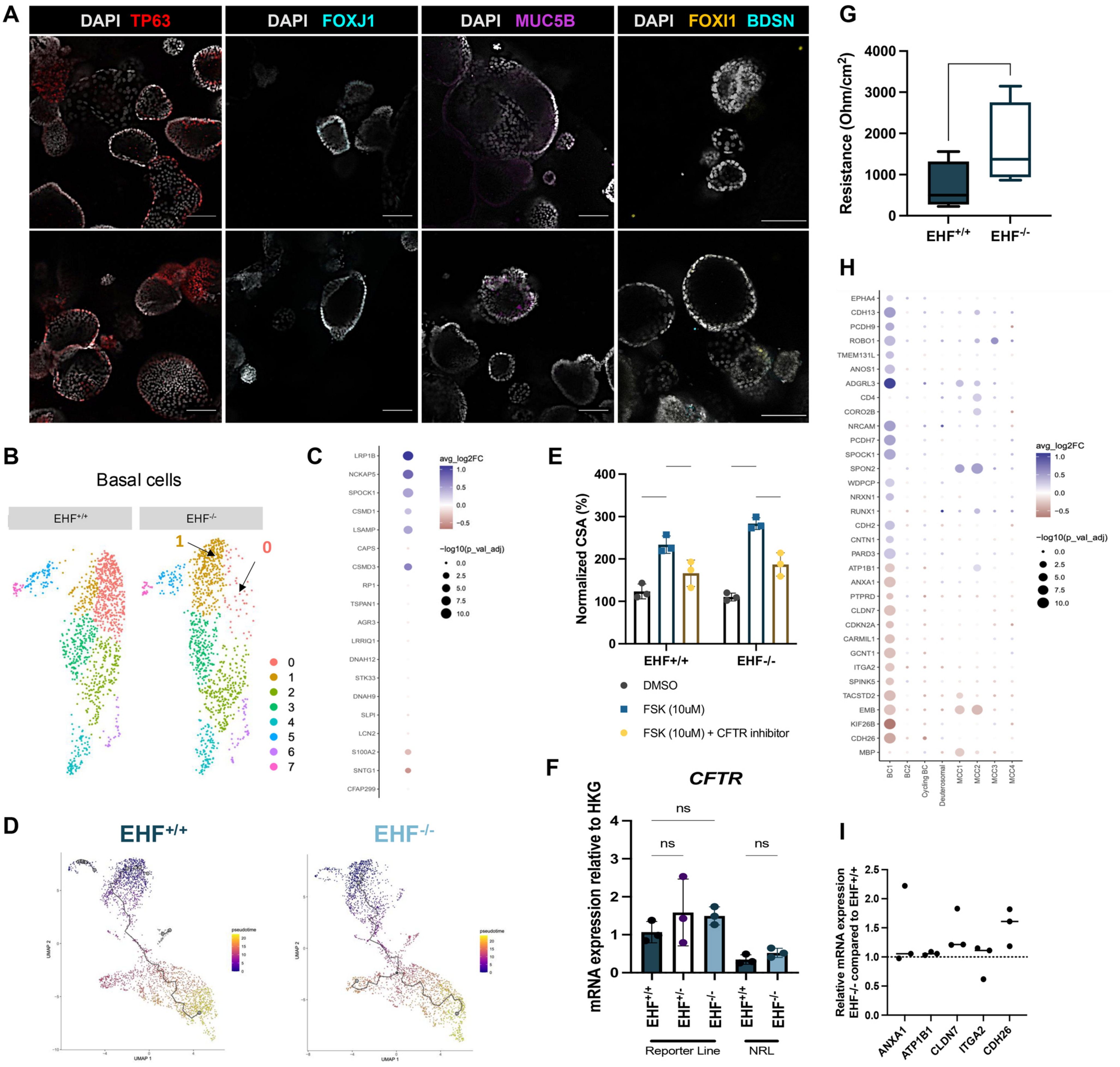

Fig. S4. Complementary characterizations of knock-out clones.

(A) Images of Day30 organoids by confocal microscopy immunolabeled with antibodies (scale bar=100 um). **(B)** UMAP visualisation of EHF$^{+/+}$ or EHF$^{-/-}$ cells basal cell population (scRNAseq, Day60 cells, Reporter line) colored by cluster assignment. **(C)** Dot plot of genes differentially expressed in subcluster 0 compared to subcluster 1 of Day60 basal cells. Blues represent genes expressed more in subcluster 0. **(D)** UMAP visualization of pseudotimes courses from cycling basal cells for EHF$^{+/+}$ or EHF$^{-/-}$ cells. **(E)** Sweeling assay on Day30 organoids treated by either vehicle (DMSO) or 10uM forskolin (FSK), or or 10uM forskolin (FSK) + CFTR inhibitor for 24h (Reporter line, n=3 experimental replicates. Error bars represent SD. Statistical significance: one-way anova test; *P < 0.05, ; **P < 0.005, ****P < 0.00005). **(F)** Gene expression of *CFTR* by RTqPCR of Day30 cells (n=3 experimental replicates per line. Reporter Line and non-reporter line. Error bars represent SD. Statistical significance determined by paired, one-way anova). **(G)** TEER measured with a voltammeter. (NRL, n=4 experimental replicates, unpaired t-test, *P < 0.05). **(H)** Dot plot of genes involved in tight junctions differentially expressed in EHF$^{+/+}$ compared to EHF$^{-/-}$ cells. Blues represent genes expressed more in EHF$^{+/+}$. **(I)** Relative expression of EHF$^{-/-}$ cells compared to EHF$^{+/+}$ cells by RTqPCR of genes outlined by scRNAseq.

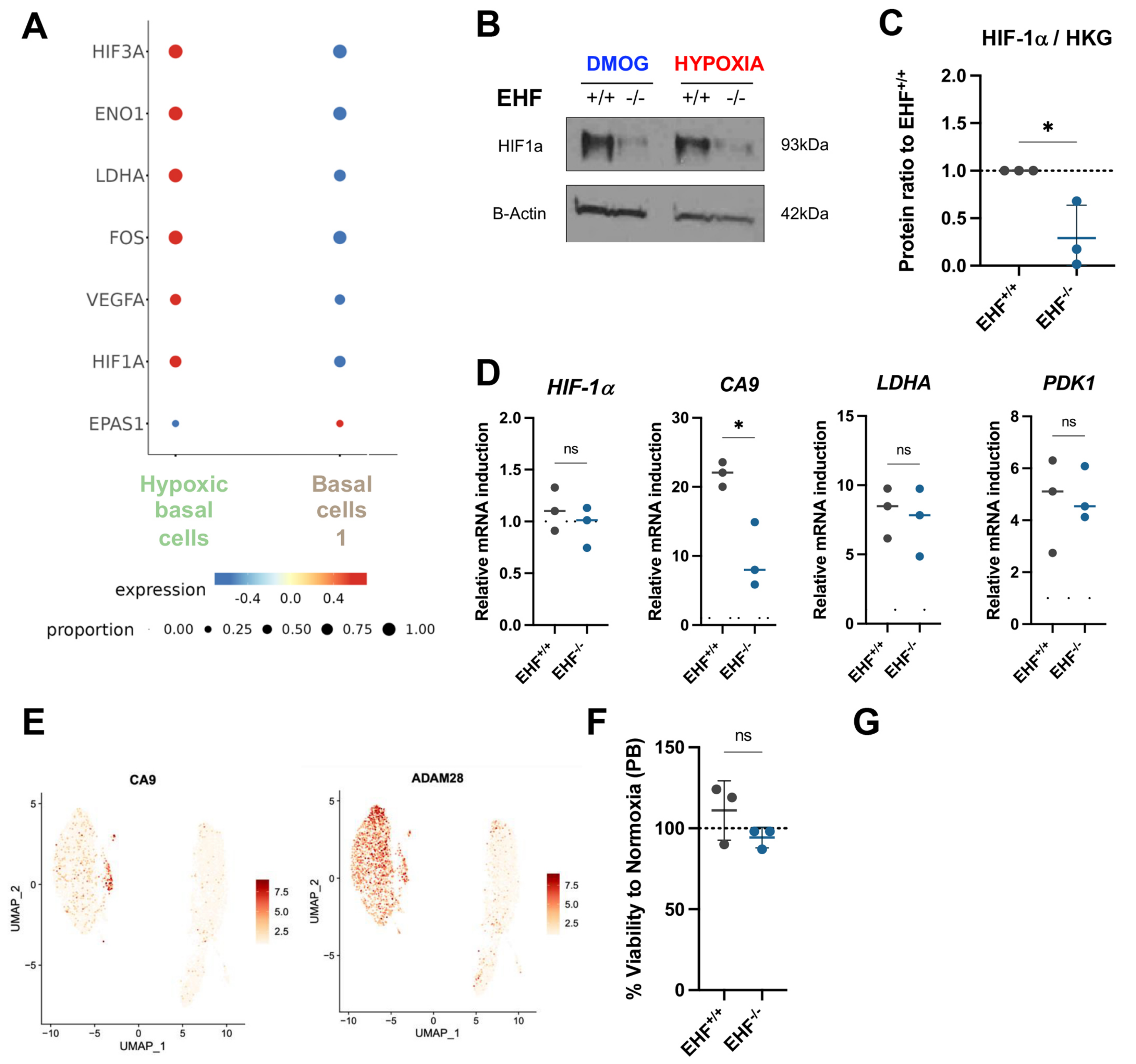

Fig. S5. Complementary phenotypes of experiments (Day 60 cells) in hypoxia.

(A) Dot plot showing expression of marker genes differentiating clusters 1 and 2 at resolution 0.04. **(B)** Representative WB of HIF-1α after 24h of treatment by DMOG or 1% hypoxia. ß-actin as loading control. Non-reporter Line. **(C)** Quantification (n=3 experimental replicates. Non-reporter Line. Error bars represent SD. Statistical significance determined by paired, one-way anova; *P < 0.05). **(D)** Gene expression induction by RTqPCR of Day60 cells after an incubation in 1% O_2 for 24h (n=3 experimental replicates. Non-reporter Line. Error bars represent SD. Statistical significance determined by paired, one-way anova; *P < 0.05). **(E)** UMAP visualisations of expression for CA9 and ADAM28. **(F)** Viability of cells compared to normoxic controls by Prestoblue assay after 24h in hypoxia (1% O_2) **(G)** Representative WB of CA9 after treatment by DMOG or 1% hypoxia for 24h. Histone 3 (H3) as loading control.

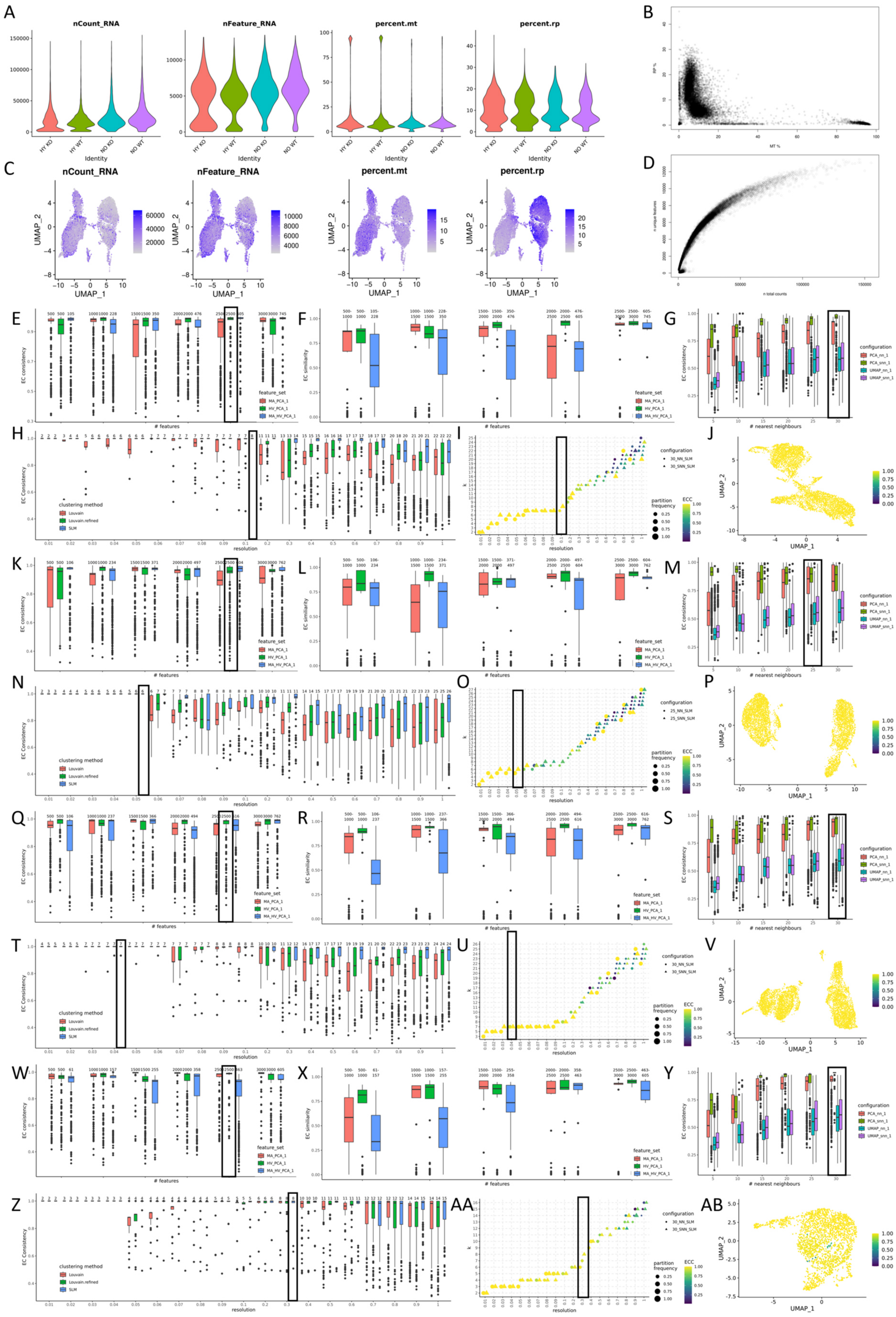

Disease Models & Mechanisms • Supplementary information

Fig. S6. Quality control of scRNAseq data for all samples. A-D) Quality control overview, E-J) ClustAssess analysis for normoxia samples, K-P) ClustAssess analysis for hypoxia samples, Q-V) ClustAssess analysis for wild type samples, W-AB) ClustAssess analysis for normoxia basal cells
(A) Violin plots showing distribution of nFeature, nCount, %MT, %RP before filtering, per sample. (B) Scatter plot comparing %MT and %RP for each cell before filtering. (C) Distribution of nFeature, nCount, %MT and % RP on UMAP on all samples after filtering out low quality cells. (D) Saturation scatter plot comparing nFeature and nCount for each cell before filtering For panels E-AB, the selected settings are highlighted using a black box. (E-K-Q-W) Boxplots showing distribution of element-centric clustering consistency when the PCA/ UMAP is calculated on incremental numbers of most abundant genes, highly variable genes or the intersection of those sets. The number of genes used in each case is displayed above the boxplot. The stability assessment is performed across 30 runs, each with different random seeds, on the three gene sets described. Higher element-centric clustering consistencies correspond to more stable clusterings. In each case, the top 2500 highly variable genes were selected. (F-L-R-X) Boxplots showing distribution of element-centric clustering similarity comparing the most frequent partitions of consecutive steps, using most abundant genes, highly variable genes or the intersection of those sets. The aim of this is to determine when adding more genes moves from the signal to the noise zone. (G-M-S-Y) Boxplots showing distribution of element-centric clustering consistency using different numbers of neighbours to compute cell adjacencies, and comparing SNN vs NN graphs. In each case, the shared nearest neighbour graph was selected as the more stable option, using 30 neighbours in each case apart from in hypoxia where 25 was a more stable option. (H-O-T-Z) Distribution of element-centric clustering consistency (ECC) comparing different clustering algorithms (Louvain, Louvain-refined, SLM) and resolution settings (between 0.01 and 1), across partition lists obtained over multiple runs using varied random seeds. Above each boxplot, the number of clusters using that clustering algorithm and resolution is displayed. Compared to Louvain and Louvain-refined, SLM generally demonstrates higher stability and was selected in each case. The resolutions selected are highlighted by a black box for each subset of the data. (I-P-U-AA) Scatter plot showing relationship between number of clusters and resolution using SLM clustering algorithm. The colour gradient represents either the element-centric clustering consistency of clusterings obtained using that resolution. of them. Lighter (higher) values show high stability with respect to changes of the random seed. The size reflects the frequency of the most common partition using fixed resolution and number of clusters, another approximation of the stability of the clustering. The shared nearest neighbour graph was selected in every case and the selection of resolutions using panels H/O/T/Z is confirmed by this analysis. (J-Q-V-AB) Selected UMAP representation coloured by element-centric clustering consistency for the selected clustering settings. Any areas with darker colours show lower stability with respect to the changing of random seeds.

Disease Models & Mechanisms • Supplementary information

Table S1. Media composition

Name	Compound
hiPSC Medium	500ml DMEM/F12 (Life Technologies)<br>10ml ITS (Life Technologies)<br>3.6ml Sodium bicarbonate 7.5% (Life Technologies)<br>5ml LAA (Life Technologies)<br>5ml Peni/Strep (Life Technologies)
Lung Base Medium	Advanced DMEM/F12 (Life Technologies)<br>2.5ml Glutamax (Life Technologies)<br>1.95ml LAA (Life Technologies)<br>8.6ul MTG (Sigma)<br>1% Peni/Strep (Life Technologies)<br>1% B27 (Life Technologies)
Definitive Endoderm#1	250ml F-12 (Life technologies)<br>250ml IMDM (Life technologies)<br>Concentrated Lipids (Life technologies)<br>5ul MTG (Sigma)<br>250ul Transferrin (Sigma)<br>350ul Insulin (Roche)<br>5ml Penicillin-Streptomycin (Life technologies)<br>0.5g PVA (Sigma)
Definitive Endoderm#2	RPMI (Thermo Fisher) with:<br>2% B27 (Life Technologies)<br>1% NEAA (Gibco)<br>1% Penicillin-Streptomycin (Life technologies)

Disease Models & Mechanisms • Supplementary information

Table S2. Sequences of primers and gRNA used

Experiment	Gene	RTqPCR primer sequences (Forward / Reverse)
RT-qPCR	*UBC*	ATTTGGGTCGCGGTTCTTG / TGCCTTGACATTCTCGATGGT
	PBGD	GGAGCCATGTCTGGTAACGG / CCACGCGAATCACTCTCATCT
	RPLPO	GGCGTCCTCGTGGAAGTGAC / GCCTTGCGCATCATGGTGTT
	OCT4	AGTGAGAGGCAACCTGGAGA / ACACTCGGACCACATCCTTC
	SOX2	TGGACAGTTACGCGCACAT / CGAGTAGGACATGCTGTAGGT
	SOX17	CGCACGGAATTTGAACAGTA / GGATCAGGGACCTGTCACAC
	FOXA2	AGGAGGAAAACGGGAAAGAA / CAACAACAGCAATGGAGGAG
	NKX2.1	ACCAGGACACCATGAGGAAC / CGCCGACAGGTACTTCTGTT
	TP63	ACCCTATTGCTTTTAGCCTCCC / GTGGAATACGTCCAGGTGGC
	KRT5	GGAGTTGGACCAGTCAACATC / TGGAGTAGTAGCTTCCACTGC
	NGFR	CCTGGACAGCGTGACGTTC / CCCAGTCGTCTCATCCTGGT
	FOXJ1	GAGCGGCGCTTTCAAGAAG / GGCCTCGGTATTCACCGTC
	MUC5AC	GCACCAACGACAGGAAGGATGAG / CACGTTCCAGAGCCGGACAT
	MUC5B	ACCAGAACCAGGCTGACGAC / TGCCGGGCGTAGTTCTCATT
	CFTR	CTATGACCCGGATAACAAGGAGG / CAAAAATGGCTGGGTGTAGGA
	SCGB3A2	CCTTGTGGAGGGGCTAAGGAA / CACCAAGTGTGATAGCGCCT
	SCGB1A1	TCATGGACACACCCTCCAGTTATGAG / TGAGCTTAATGATGCTTTCTCTGGGC
	MUC4	GCCCAAGCTACAGTGTGAACTCA / ATGGTGCCGTTGTAATTTGTTGT
	MUC20	AGAGTGGCAGAAAGGCTGATGC / CTGATGTCCGTTAGCCTCTCCT
	EHF	ATCTGAAGTGGAACGGCCAG / GTCATGGAGATCTGAGCCCG
	APIP	GCGCAGGACAAGGAGCAT / TTCTTCGATGGCGAAGGTCC
	SLC9A3	CACCGTCATCTTCCAGTGGC / GGATATGTCCTCGATGGCCG
	EXOC3	ACCGGAAAAAGCAAACTGGC / TGCCCTCAATTCTGGTGGTC
	SLC6A14	GTGACTCAGGCTGGAATTTACT / TATCCACCTCTTTGCTCCAATC
	ß-Actin	CTGGGAGTGGGTGGAGGC / TCAACTGGTCTCAAGTCAGTG
	HIF-1a	CCAGTTACGTTCCTTCGATCAGT / TTTGAGGACTTGACTTGCGCTTTCA
	HIF-2a	AAGCCTTGGAGGGTTTCATTG / TGCTGATGTTTTCTGACAGAAA
	CA9	GCCGCCTTTCTGGAGGA / TCTTCCAAGCGAGACAGCAA
	LDHA	CCATGATTAAGGGTCTTTACGG / GGTCTGAGATTCCATTCTGTCC
	PDK1	ATGTACCATCCCATCTCTATCAC / GGTCACTCATCTTCACAGTC
qPCR (genotyping)	EHF, Exon#3	TCATCCAGTGTCCAGGGGTA / ACACCAGGTACCCTATGCTTC
gRNA	gRNA#1	TCAGTACTGGACCAAGTACCAGG
	gRNA#2	ACTGGACCAAGTACCAGGTGTGG

Disease Models & Mechanisms • Supplementary information

Table S3. Antibodies used for IF, flow cytometry, and cell sorting

Antibody	Species	Distributor	Cat#	Dilution
NKX2.1 (TTF1)	Mouse	Abcam	ab242428	1:200
TP63	Rabbit	Abcam	ab124762	1:200
KRT5	Mouse	Abcam	ab17130	1:200
NGFR-BV421	Mouse	BD Biosciences	562562	1:200
ACT	Rabbit	NEB	5335S	1:400
FOXJ1	Mouse	Abcam	ab20454	1:200
SCGB3A2	Goat	Abcam	ab181853	1:200
SCGB1A1	Mouse	Santa Cruz	sc-365992	1:200
MUC5AC	Mouse	Thermofisher	MA1-38223	1:200
CPM	Mouse	Fujifilm	014-27501	1:200
Anti-Mouse AF488	Mouse	Life Technologies	A-21202	1:1000
Anti-Mouse AF568	Mouse	Life Technologies	A-10037	1:1000
Anti-Rabbit AF568	Rabbit	Life Technologies	A-10042	1:1000
Anti-Goat AF647	Goat	Life Technologies	A-21447	1:1000
β-actin	Mouse	Sigma	A2228	1:200
EHF	Rabbit	Thermofischer	PA5-84038	1:200
HIF1a	Rabbit	Cell Signalling	36169	1:1000
HIF2a	Rabbit	Cell Signalling	7096	1:1000
CA9	Mouse	AbsoluteAntibody	Ab00414-1.1	1:5000
FOXI1	Goat	Abcam	Ab20454	1:200
BSND	Rabbit	Abcam	Ab238741	1:200
MUC5B	Rabbit	Abcam	XXAb87376	1:200
